# Efficacy and safety of guanxinshutong capsule as adjunctive therapy for unstable angina: an integrated systematic review, meta-analysis, and network pharmacology study

**DOI:** 10.3389/fcvm.2026.1740334

**Published:** 2026-02-24

**Authors:** Ya Li, Liyuan Yu, Lulu Wu, Weihang Peng, Qingmin Li, Peiying Huang, Xiaohui Chen, Ye Ye, Bojun Chen, Li Chen

**Affiliations:** 1 The Second Clinical Medical College, Guangzhou University of Chinese Medicine, Guangzhou, China; 2 Cardiology Department, The First People's Hospital of Chenzhou, Chenzhou, China; 3 Emergency Department, The Second Affiliated Hospital, Guangzhou University of Chinese Medicine, Guangzhou, China

**Keywords:** unstable angina, guanxinshutong capsule, efficacy and safety, meta-analysis, network pharmacology analysis

## Abstract

**Background:**

Unstable angina (UA), characterized by worsening chest pain and increased risk of acute myocardial infarction or sudden death, is a major clinical condition necessitating urgent and effective intervention. Although guanxinshutong capsule (GXST) has demonstrated preliminary therapeutic potential in alleviating angina symptoms, it lacks sufficient and robust clinical evidence to confirm its efficacy and safety in UA treatment. Therefore, further clinical research is urgently needed to validate the practical value of GXST in managing UA.

**Objective:**

To determine the efficacy and safety of GXST as an adjunctive therapy for UA and to elucidate its potential pharmacological mechanisms.

**Methods:**

Relevant RCTs were included to investigate the effectiveness of GXST in combination with WM for UA. ROB 2 was applied to assess their methodological quality. The data integration, evidence quality assessment, and trial sequence analysis were performed using R software, the GRADE framework, and TSA software, respectively. Concurrently, the network pharmacology was employed to identify disease-relevant targets, active components, and core targets of GXST. Crucially, bioinformatics analysis was conducted to explore the potential regulatory mechanisms.

**Results:**

Fifteen RCTs were included. Compared with WM monotherapy, GXST combined with WM exhibited significantly superior efficacy across multiple indicators: clinical effective rate(RR = 1.19, 95% CI = 1.13–1.25), ECG effective rate (RR = 1.20, 95% CI = 1.07–1.34), angina frequency (SMD = −2.20, 95% CI = −3.36 to −1.04), angina duration (SMD = −1.54, 95% CI = −2.14 to −0.94), PV levels(SMD = −0.82, 95% CI = −1.23 to −0.41), FIB levels(SMD = −1.18, 95% CI = −1.50 to −0.86), and TCM syndrome scores (SMD = −1.68, 95% CI = −2.18 to −1.18). However, no significant intergroup differences were detected in CK-MB, cTnI, or ARDI. KEGG enrichment analysis highlighted the PI3K-Akt and MAPK signaling pathways as central to the underlying mechanism. Molecular docking further demonstrated pronounced binding affinities of kaempferol, miltirone, and asiatic acid toward core targets AKT1, MAPK3, and PIK3CA, corroborating their therapeutic potential.

**Conclusion:**

The combination therapy of GXST and WM significantly boosted clinical efficacy in patients with UA. Its mechanism of action involves regulating the PIK3CA/AKT1 and MAPK3 signaling pathways.

**Systematic Review Registration:**

https://www.crd.york.ac.uk/PROSPERO/view/CRD42025634213, PROSPERO CRD42025634213.

## Graphical Abstract

**Figure:**
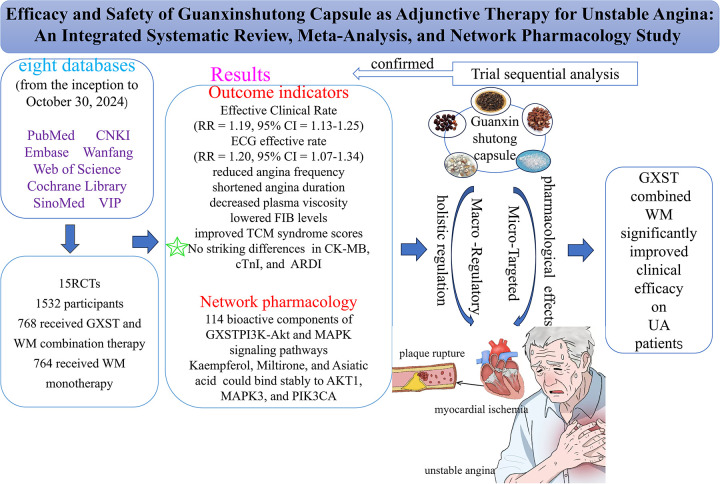
Graphical abstract of the meta-analysis .

## 1 Introduction

UA, a prevalent subtype of acute coronary syndrome (ACS), is defined by severe, rest-induced, and prolonged angina pectoris (1, 2), the pathogenesis of which primarily involves myocardial ischemia resulting from focal coronary artery lesions and plaque rupture with subsequent thrombosis (3, 4). Epidemiologically, UA accounted for 38.1% of coronary heart disease (CHD)-related hospital admissions in China in 2022 (5); in the United States alone, it resulted in approximately 18,000 hospitalizations in 2019 (6). Clinically, UA presents with progressive chest pain and confers a high risk of progression to acute myocardial infarction or sudden cardiac death, thereby representing a major contributor to mortality in patients with CHD (3, 7).

The management of UA relies on pharmacological and non-pharmacological interventions to alleviate angina episodes, improve patients' quality of life, and decrease mortality (4). Pharmacological treatments primarily include antiplatelet agents, nitrates, β-blockers, and lipid-lowering medications (8), with recent meta-analyses indicating that antiplatelet therapies like aspirin are associated with a significant risk of gastrointestinal bleeding and thrombotic events, underscoring the necessity for careful risk-benefit assessment in clinical practice (9, 10). While non-pharmacological treatments include interventional therapies, smoking cessation, and dietary adjustments (11), exercise-based cardiac rehabilitation has been shown to reduce cardiovascular mortality by up to 26% and hospitalizations by 23% in patients with CHD, underscoring its role as a core component of comprehensive care (12). Despite effectively stabilizing disease progression, these interventions have some limitations. For example, antiplatelet drugs pose a substantial risk of both bleeding and thrombotic events (13), nitrates tend to induce headaches and hypotension (14), and β-blockers may cause atrioventricular block and bradycardia (15). Moreover, invasive procedures like percutaneous coronary intervention (PCI) increase the risk of thrombosis and arrhythmias (16). Although these interventions improve prognosis in most patients, their safety issues and limited use in specific populations highlight the need for novel adjuvant therapies to impede disease progression and reduce mortality. Growing evidence suggests that traditional Chinese Medicine (TCM) is a valuable adjuvant therapy for UA, with studies demonstrating that TCM formulations like Ginkgo biloba extract can significantly improve health-related quality of life and reduce angina symptoms in UA patients, offering a complementary approach with minimal adverse effects (17).

GXST, a classic TCM formula, is famous for promoting blood circulation and resolving stasis, mitigating meridian obstruction, and activating qi flow to relieve pain. Clinically, it has been used for decades to manage CHD, angina pectoris, and heart failure (18, 19). It comprises five TCM components: *Choerospondias axillaris (Roxb.)* B.L. Burtt & A.W. Hill, *Salvia miltiorrhiza* Bunge, *Syzygium aromaticum (L.)* Merr. & L.M.Perry, *Blumea balsamifera (L.)* DC, and *Bambusa textilis* McClure (20). Pharmacological studies revealed that GXST regulates lipid metabolism, inhibits myocardial apoptosis, and modulates myocardial energy metabolism (21).

Previous studies have indicated that GXST alleviates UA symptoms, inhibits disease progression, and improves prognosis by promoting coronary dilation, ameliorating myocardial ischemia, and maintaining plaque stability (22). A meta-analysis demonstrated that the combination of GXST and WM is more effective for UA than WM alone (23). However, this meta-analysis had some limitations: several included studies failed to isolate GXST as the sole intervention variable in experimental groups (24–26), and it incorporated an RCT on GXST for stable angina pectoris (27). Both of these issues weakened the credibility of the conclusions. Furthermore, the previous study was limited to clinical efficacy and omitted the pharmacological mechanisms of GXST. To address these, we conducted an updated, rigorous meta-analysis including recent studies and more outcome measures to improve the reliability of the clinical evidence. Beyond clinical evaluation, we incorporated network pharmacology and molecular docking to systematically elucidate GXST's multi-target mechanisms against UA, a method rarely used in studying Chinese patent medicines for cardiovascular diseases. This approach helps validate GXST's benefits, bridge traditional and modern perspectives, and support its standardized clinical use.

## 2 Methods

The study was prospectively registered in PROSPERO (registration number CRD42025634213) following the PRISMA guidelines and its extension statement (28, 29). The PRISMA checklist was presented in Supplementary Appendix 1.

### 2.1 Search strategy

To retrieve randomized controlled trials (RCTs) assessing the effectiveness of GXST in combination with WM for UA, eight databases [PubMed, Embase, China National Knowledge Infrastructure (CNKI), Cochrane Library, Web of Science, VIP, Wanfang, and SinoMed] were searched comprehensively. As of November 30, 2024, the bilingual literature search has been conducted in both Chinese and English. The search scope encompassed controlled vocabulary terms such as “unstable angina”, “Guanxinshutong capsules”, and “angina pectoris”, supplemented by free-text keywords. Detailed search strategies and specific results are provided in the supplementary materials (Supplementary Appendix 2, Supplementary Tables 1–5).

### 2.2 Selection criteria

#### 2.2.1 Inclusion criteria

1. Research population: Participants diagnosed with UA based on established clinical criteria (30–32).
2. Intervention protocols: The control group received standard WM therapy for UA according to contemporary treatment guidelines (33, 34). The experimental group was provided with the GXST and a similar standard WM intervention to the control group.
3. Study design: RCTs.
4. Outcome measures: Patients meeting any of the following outcome indicators were incorporated in the analysis.

**Primary Outcome Indicators:**

1. Clinical Effective Rate:

This indicator was designed to estimate the therapeutic efficacy for angina pectoris according to the unified *Guidelines for Clinical Research on New Traditional Chinese Medicines* (GCRNCM). Marked efficacy was determined by complete symptom resolution, accompanied by a significant decrease in the frequency, severity, and duration of angina pectoris. Partial efficacy was characterized by partial symptom improvement. Cases exhibiting no substantial amelioration or deterioration of symptoms were classified as Inefficacy (35). The total clinical effective rate was calculated as: (number of cases with marked efficacy + number of cases with partial efficacy)/total number of cases × 100%.

2. ECG Effective Rate:

This parameter quantified the improvement of myocardial ischemia on electrocardiography. Consistent with the unified standards of GCRNCM, this indicator was defined as the proportion of patients whose abnormal ECG manifestations (e.g., ST-segment depression, T-wave inversion) returned to normal or showed significant improvement compared with baseline.

3. **Characteristics of the Angina Pectoris Episode:**
  A meticulous comparison of angina pectoris frequency and duration was conducted for patients with UA, contrasting pre-intervention baseline with post-intervention assessments.

**Secondary Outcome Indicators:**

4. **Hemodynamic and Cardiac Biomarkers:** plasma viscosity (PV), fibrinogen (FIB), and cardiac-specific biomarkers (e.g., cardiac troponin I(cTnI) and Creatine Kinase-MB(CK-MB) were quantitatively measured pre-and post-therapy.
5. TCM Syndrome Scores:

The TCM Syndrome Scores were established in alignment with GCRNCM (35). For patients with UA, primary symptoms (chest tightness, chest pain) were respectively scored 2, 4, or 6 points for mild, moderate, or severe presentations, while secondary symptoms (palpitations and dyspnea) were assigned 1, 2, or 3 points. Therapeutic outcomes were categorized as follows: Marked Efficacy (≥70% reduction in syndrome scores, achieving symptom disappearance); Partial Efficacy (30%–69% score reduction with measurable symptom improvement); Inefficacy (<30% reduction coupled with persistent or worsening symptoms).

6. Adverse Drug Reactions Incidence (ADRI):

ADRI was ascertained as the proportion of UA patients with treatment-related adverse events (dizziness, headache, gastrointestinal disorders).

#### 2.2.2 Exclusion criteria

1. Pregnant or lactating women, and patients with mental health disorders, coagulation dysfunction, or experimental drug allergies.
2. Patients with severe comorbidities (uncontrolled hypertension, severe arrhythmia, hepatic or renal insufficiency, advanced malignancies, or acute infections).
3. Duplicate publications and studies with incomplete and critically inaccurate outcome data.
4. Studies with failed randomization or significant group baseline between-group differences (36).

### 2.3 Study screening and data extraction

Eligible studies were screened and identified employing EndNote X9. Following deduplication, two investigators independently reviewed titles, abstracts, and full texts against predefined criteria. Data were extracted on the first author, publication year, randomization protocol, sample size, participant age, mean disease course, drug regimen, treatment course, and outcome measures. The rigor and reliability of the study were corroborated by cross-validation between the two reviewers, wherein any disagreements were settled through consensus discussion or by a third reviewer.

### 2.4 Quality assessment

Two researchers systematically evaluated the risk of bias in the RCTs employing the updated Cochrane Risk of Bias tool (ROB 2) (37). This assessment encompassed five critical domains: deviations from intended interventions, missing outcome data, selection of reported results, randomization process, and outcome measurements (36). Each domain was individually categorized as either “low risk”, “high risk”, or “some concerns”, predicated on established overall risk classification criteria. Discrepancies arising between reviewers were adjudicated through consensus discussion or by referral to a third independent reviewer.

### 2.5 Data analysis

All data analyses were performed by utilizing R software (Version 4.2.2) with the Metafor package. The continuous and dichotomous outcome measures were analyzed with standardized mean difference (SMD) and relative risk (RR), respectively. The statistical significance was defined as both indicators reporting their 95% confidence intervals (CI) with a *P*-value≤0.05. Heterogeneity was quantified through the *Q* test and *I*^2^ statistic, and visualized via forest plots, Labbé plots, and funnel plots. The common effects model was employed when *I*^2^ < 50% and *p* > 0.1; conversely, the random-effects model was applied. Sensitivity analysis and meta-regression were conducted to identify the sources of the heterogeneity and validate the robustness of the results. Meanwhile, significant meta-regression findings guided the subgroup analyses. Publication bias was assessed through funnel plots, Egger's, and Begg's tests for outcomes with≥10 studies. The trim-and-fill method was applied adjunctively when required to address potential bias.

### 2.6 Network pharmacology and molecular docking

Active components of GXST were retrieved from TCMSP (38) using pharmacokinetic criteria: oral bioavailability (OB) ≥30%, drug-likeness(DL) ≥0.18, and intestinal epithelial permeability (Caco-2) ≥−0.4 (39). Canonical SMILES were verified in PubChem (40). Potential therapeutic targets were predicted using SwissTargetPrediction (probability>0.1) (41) and supplemented by BATMAN-TCM (42). A compound-target interaction network was constructed to illustrate GXST's multi-component pharmacology. UA-related targets were obtained from GeneCards (score≥1.0) (43), OMIM, DrugBank, TTD, and PharmGKB. Shared targets between drug and disease were identified, visualized using a Venn diagram, and subsequently subjected to Gene Ontology (GO) and Kyoto Encyclopedia of Genes and Genomes (KEGG) enrichment analyses via the DAVID database (44). A protein-protein interaction (PPI) network was generated using the STRING database and visualized in Cytoscape 3.10.3 (45). Hub genes were identified using CytoHubba by integrating six topological algorithms: degree, closeness, maximal clique centrality (MCC), maximum neighborhood component (MNC), edge percolated component (EPC), and radiality (46).

Molecular docking was performed using AutoDock Vina (version 1.1.2) to evaluate the binding affinity and interaction patterns between bioactive components and core targets. The three-dimensional structures of the key target proteins were retrieved from the RCSB Protein Data Bank (PDB), including the crystal structures of AKT1 (PDB ID: 3OS5), MAPK3 (PDB ID: 6GES), and PIK3CA (PDB ID: 2RD0). The chemical structures of the ligands were downloaded from the PubChem database. Before docking, all water molecules and non-essential ligands were removed from the protein structures using PyMOL 3.0.3, and polar hydrogens were added with AutoDock Tools 1.5.7. Both protein and ligand files were converted into the PDBQT format. The grid box was centered on the active site of each target (size: 40 Å × 40 Å × 40 Å; spacing: 0.375 Å). The docking search space was defined accordingly, and the exhaustiveness parameter was set to 8. The conformation with the lowest binding energy (most negative value) was selected for further interaction analysis.

## 3 Results

### 3.1 Study screening

**Figure 1:**
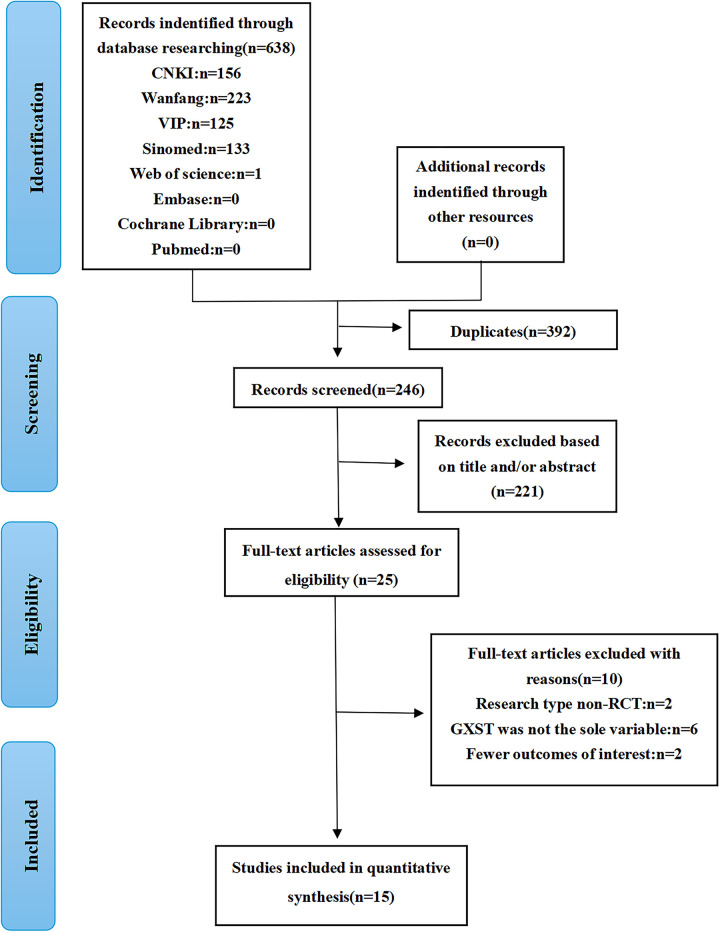
The flowchart of the literature screening process.

An initial search retrieved 638 relevant articles. After titles, abstracts, and full-text screening, 15 articles (22, 47–60) involving 1,532 patients were ultimately included. The flowchart of the literature screening is presented in Figure 1; Table 1 summarizes the studies' baseline characteristics.

**Table 1:** The baseline characteristics of the included studies.

Study ID	Stochastic Mode	Sample size(M/F)		Age(years)		Course of Disease(years)		Interventions		Duration of therapy	Outcome indicators
		Treatment group	Control group	Treatment group	Control group	Treatment group	Control group	Treatment group （GXST + WM）	Control group (WM)		
Huang and Li (2023) (52)	Random number table method	20/19	17/22	58.64 ± 3.27	58.43 ± 3.31	3.50 ± 1.27	3.57 ± 1.25	GXST + WM (0.9 g po, tid)	Metoprolol tartrate capsule 25 mg po, tid	eight weeks	③④⑤
Qin and Xiao (2022) (22)	ND	31/26	32/25	65.27 ± 3.28	65.77 ± 3.49	4.03 ± 1.49	4.51 ± 1.61	GXST + WM (0.9 g po, tid)	Ticagrelor tablets 90 mg po, bid	eight weeks	①④
Gou and Zhang (2020) (51)	Random number table method	76/74	72/78	53.81 ± 8.12	52.48 ± 7.29	ND	ND	GXST + WM (0.9 g po, tid)	Metoprolol tartrate tablets 25 mg po, tid	four weeks	①③④⑥
Song (2020) (53)	ND	24/19	22/21	62.15 ± 7.61	61.97 ± 7.50	4.25 ± 1.60	4.35 ± 1.58	GXST + WM (0.9 g po, tid)	β-receptor blocker, Anti-platele drugs, Nitrates, CCB, 40 mg db-cAMP + 250 mL NS igvtt, qd	four weeks	③⑥
Zhao (2020) (54)	NS	22/20	20/22	68.0 ± 1.8	68.2 ± 2.0	ND	ND	GXST + WM (0.3 g po, tid)	Diltiazem controlled release tablet 30 mg po, tid	ND	③
Du (2019) (56)	ND	24/13	23/13	68.4 ± 5.2	67.7 ± 5.8	6.2 ± 4.1	6.5 ± 4.2	GXST + WM (0.9 g po, tid)	Aspirin 100 mg po, qd; HMWH 0.4 mg ih, bid; Nitrates 40 mg po, qd; ACEI 40 mg po,qd	four weeks	①②
Chen and Cao (2018) (50)	ND	29/23	27/25	58.7 ± 3.6	59.2 ± 4.2	5.1 ± 1.4	5.3 ± 1.6	GXST + WM (0.9 g po, tid)	Nitrates, β-receptor blocker, CCB、Anti-platelet drugs, Simvastatin tablet 20 mg po, qd	four weeks	①②③⑥
Miao and Cheng (2017) (58)	NS	27/17	25/18	55.90 ± 7.29	56.88 ± 7.32	5.10 ± 1.30	4.17 ± 1.21	GXST + WM (0.9 g po, tid)	ISMN 5 mg po, tid; Atorvastatin calcium tablets 10 mg po, qd	four weeks	②③④⑥
Lin (2016a) (61)	Random number table method	21/18	23/16	65.13 ± 7.04	64.81 ± 6.97	24.12 ± 3.85	23.83 ± 3.61	GXST + WM (0.9 g po, tid)	Aspirin, β-receptor blocker, Nitrates, Statins, ACEI	four weeks	①
Chen et al. (2016b) (49)	Random number table method	21/29	24/26	53.0 ± 14.0	54.0 ± 14.8	6.0 ± 3.5	6.0 ± 3.2	GXST + WM (0.9 g po, tid)	Nitrates, Antiplatelet drugs, β-receptor blocker; LMWH-Ca, Statins	twelve weeks	①③
Chen et al. (2016) (55)	Random number table method	21/29	24/26	53 ± 13	54 ± 14	6 ± 3	6 ± 3	GXST + WM (0.9 g po, tid)	ISDN、LMWH-Ca, β-receptor blocker, Anti-platelet drugs, CCB Trimetazidine hydrochloride 20 mgpo, tid	four weeks	①③⑥
Zhang (2015) (59)	ND	23/17	21/19	61 ± 5.5	64 ± 6.2	ND	ND	GXST + WM (0.9 g po, tid)	β-receptor blocker, Nitrates, statins, CCB, ACEI、Antiplatelet drugs	four weeks	①⑥
Zhao and Zhang (2015) (57)	NS	24/16	22/18	55.45 ± 8.35	56.86 ± 9.04	3.35 ± 1.07	3.67 ± 1.58	GXST + WM (0.9 g po, tid)	Aspirin 0.1 g po, qd; ISMN 50 mg po, qd; Atorvastatin 10 mg po, qd; Diltiazem hydrochloride 30 mg po, tid	four weeks	①②
Liang (2015) (47)	NS	27/16	25/18	61.8 ± 4.2	61.1 ± 5.6	ND	ND	GXST + WM (0.9 g po, tid)	β-receptor blocker, Nitrates ACEI, Statins, Platelet inhibitors	four weeks	①⑤
Guo and Li (2013) (48)	NS	23/19	22/18	63.42 ± 17.73	62.21 ± 16.34	5.41 ± 3.27	5.52 ± 3.43	GXST + WM (0.9 g po, tid)	β-receptor blocker, Nitrate, ACEI, Statins, Platelet inhibitors	four weeks	②⑤⑥

M, males; F, females; GXST, guanxinshutong capsule; WM, Western medication; CCB, calcium channel blocker; ACEI, angiotensin-converting enzyme inhibitors; ISDN, isosorbide dinitrate; LMWH, low molecular weight heparin; ISMN, isosorbide mononitrate; ND, not described; NS, not specified; ①, clinical effective rate; ②, ECG effective rate; ③, characteristics of the angina pectoris episode; ④, hemodynamic and cardiac biomarkers; ⑤, TCM syndrome scores; ⑥, adverse drug reactions incidence (ADRI).

The NCT registration numbers for these studies were not accessible on ClinicalTrials.gov.

### 3.2 Assessment of study quality

**Figure 2:**
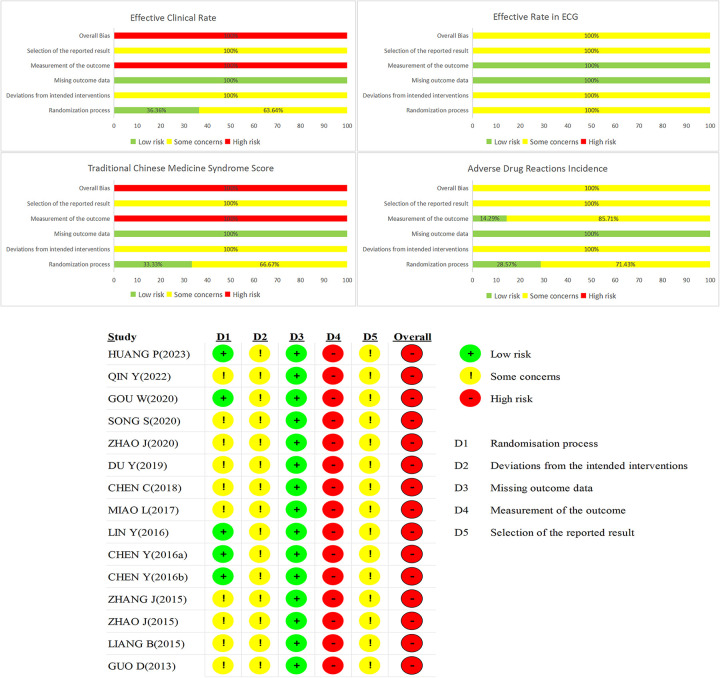
Assessments of risk bias.

One-third of the RCTs (33.33%) were rated “low risk” for using random number tables (49, 51, 52, 55, 60). An equal number (33.33%) were rated “high risk” for failing to describe the randomization method, while the remaining RCTs were rated as having “some concerns” for only stating “randomization” without elaboration (47, 48, 54, 57, 58). No studies described allocation concealment or blinding procedures, resulting in an overall rating of “some concerns” for these domains. Similarly, the lack of pre-specified protocols and insufficient information led to “some concerns” regarding deviations from intended interventions and selective reporting. “Missing outcome data” was graded “low risk” as no trials had incomplete data. However, the use of subjective outcome measures (e.g., angina and TCM scores) was considered “high risk”. Consequently, the overall risk of bias across the included RCTs was high. Risk of bias assessments are summarized in Supplementary Appendix 3; Figure 2.

### 3.3 Meta-analysis results

#### 3.3.1 Primary outcome indicators

##### 3.3.1.1 Clinical effective rate

**Figure 3:**
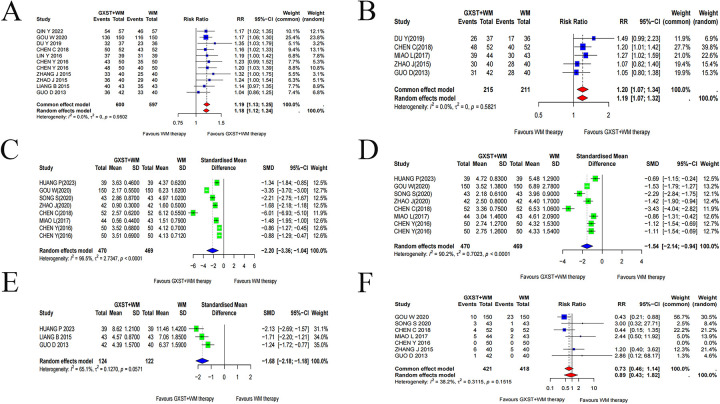
Forest plots for meta-analysis. **(A)** Clinical effective rate; **(B)** ECG effective rate; **(C)** frequency of angina pectoris; **(D)** duration of angina pectoris; **(E)** TCM syndrome scores; **(F)** adverse drug reaction incidence (ADRI).

Eleven studies reported the clinical response rate (22, 47–51, 55–57, 59, 60). Given the low heterogeneity (*I*^2^ = 0%, *P* = 0.9502) (Supplementary Appendix 4), a fixed-effect model was applied. The results revealed that combination therapy with GXST and WM significantly alleviated angina symptoms (RR = 1.19, 95% CI = 1.13–1.25) (Figure 3A).

##### 3.3.1.2 ECG effective rate

Five studies (48, 50, 56–58) reported the ECG effective rate. Due to the low heterogeneity (*I*^2^ = 0%, *P* = 0.5821), a fixed-effect model was applied. The findings indicated a significantly higher ECG effective rate in the experimental group (RR = 1.20, 95% CI = 1.07–1.34) (Figure 3B).

##### 3.3.1.3 Characteristics of the angina pectoris episode

Eight RCTs reported angina frequency (49–55, 58). Given the significant heterogeneity (*I*^2^ = 96.5%, *P* < 0.0001), a random-effects model was used. The results indicated that GXST combined with WM significantly decreased angina frequency compared to WM alone (SMD = −2.20, 95% CI = −3.36 to −1.04). Similarly, for angina duration, high heterogeneity was observed (*I*^2^ = 90.2%, *P* < 0.0001), and the random-effects model confirmed a significant reduction in the experimental group (SMD = −1.54, 95% CI = −2.14 to −0.94) (Figures 3C,D).

#### 3.3.2 Secondary outcome indicators

##### 3.3.2.1 PV and FIB levels

Three articles (50–52) reported PV (SMD = −0.82, 95% CI = −1.23 to −0.41) and FIB levels (SMD = −1.18, 95% CI = −1.50 to −0.86), indicating that the combination therapy effectively improved hemorheological parameters (Supplementary Appendixes 5A,B).

##### 3.3.2.2 CTNI and CK-MB levels

Two studies (22, 58) measured the cTnI and CK-MB levels. No significant between-group differences were observed for cInI(SMD = −7.22, 95% CI = −16.35 to 1.90) or CK-MB levels (SMD = −4.30, 95% CI = −9.80 to 1.20) (Supplementary Appendixes 5C,D).

##### 3.3.2.3 TCM syndrome scores

Three RCTs (47, 48, 52) reported TCM syndrome scores. A random-effects model was applied based on moderate heterogeneity (*I*^2^ = 65.1%, *P* = 0.0571). The findings suggested a remarkable reduction in TCM syndrome scores compared to the control group (SMD = −1.68, 95% CI = −2.18 to −1.18) (Figure 3E).

##### 3.3.2.4 ADRI

Seven studies (48–51, 53, 58, 59) monitored adverse drug reactions. Low heterogeneity (*I^2^* = 38.2%, *P* = 0.1515) supported the use of a fixed-effect model. No statistical significance in ADRI (RR = 0.73, 95% CI = 0.46–1.14) (Figure 3F).

### 3.4 Analysis of publication bias

For the clinical effective rate, Begg's test indicated the presence of publication bias (*z* = 2.26, *p* = 0.0240), whereas Egger's test showed no evidence for it (*t* = 1.53, *p* = 0.1603). This discrepancy, along with observed funnel plot asymmetry, indicated substantial publication bias or small-study effects. The trim-and-fill method was conducted to adjust for bias, yielding minimal change in effect size (pre-adjustment RR = 1.19, 95%CI = 1.13–1.25; post-adjustment RR = 1.16, 95%CI = 1.11–1.22), supporting the robustness of the findings [Supplementary Appendix 6 (Supplementary Figure 6.1–6.2)].

### 3.5 Subgroup analyses, meta-regression, and study heterogeneity

To investigate the high heterogeneity (*I*^2^ > 50%, *P* < 0.1) observed for outcomes reported in five or more studies, we conducted a meta-regression with three covariates: treatment duration, participant age, and mean disease duration. The results showed that heterogeneity in angina frequency and duration was correlated with patient age and mean disease duration, whereas treatment duration did not (Supplementary Appendix 7). Subgroup analysis revealed reduced heterogeneity in the groups with treatment duration less than four weeks. In contrast, substantial heterogeneity persisted within subgroups stratified by participant age and disease duration, suggesting that these were not the primary sources of heterogeneity in this study [Supplementary Appendix 8 (Supplementary Figure 8.1–8.6)].

### 3.6 Sensitivity analysis

Leave-one-out sensitivity analysis confirmed that the reductions in both angina frequency and duration remained statistically significant after sequential exclusion of each study [Supplementary Appendix 9 (Supplementary Figure 9.1–9.2)]. Exclusion of the study with the lowest weight (50) substantially reduced heterogeneity (*I*^2^ = 78.5%) and significantly changed the pooled SMD and 95% CI, identifying it as a crucial source of heterogeneity [Supplementary Appendix 9 (Supplementary Figure 9.3)].

### 3.7 Grade evidence quality assessment

The evidence quality of each outcome was evaluated through the GRADEpro (https://www.gradepro.org/). Clinical efficacy rate and ADRI were rated as low quality, while the ECG response rate, angina frequency and duration, and PV were graded as very low. Ratings were downgraded due to bias risk, significant heterogeneity, limited sample sizes, and potential publication bias (Supplementary Appendix 10).

### 3.8 Trial sequential analysis

TSA (http://www.ctu.dk/tsa/) was applied to control for random errors and false-positive results in the meta-analyses for the indicators (61), such as clinical effective rate, ECG response rate, and angina frequency and duration. For the clinical effectiveness rate, the intervention group showed higher effectiveness (90.8%) than the control group (76.4%), with a relative risk reduction of 18.85%. The cumulative Z-curve crossed both the conventional significance threshold (*Z* = 1.96) and the required information size (RIS = 208) after the third trial, confirming the superiority of GXST combined with WM over WM alone in treating UA. The fact that the cumulative sample size exceeded the RIS further confirmed the robustness of the findings. With an adjusted *λ* = 2, the Z-curve again surpassed the RIS and traditional significance threshold (*Z* = 1.96) after the fourth study, further validating the conclusion. TSA trajectory plots are presented in [Supplementary Appendix 11 (Supplementary Figure 11.1–11.5)].

### 3.9 Network pharmacology analysis

**Figure 4:**
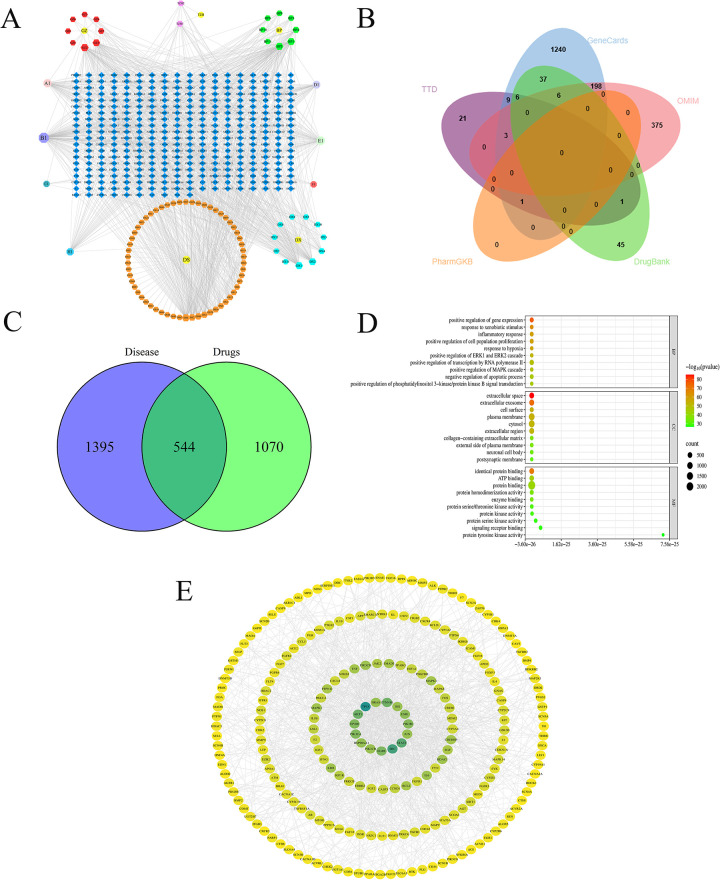
The results of network pharmacology. **(A)** Correspondence of GXST ingredients and targets; **(B)** Venn analysis of disease targets from five databases; **(C)** Venn analysis of drug targets and disease targets; **(D)** KEGG enrichment analyses; **(E)** PPI network of drug-disease interactions.

**Figure 5:**
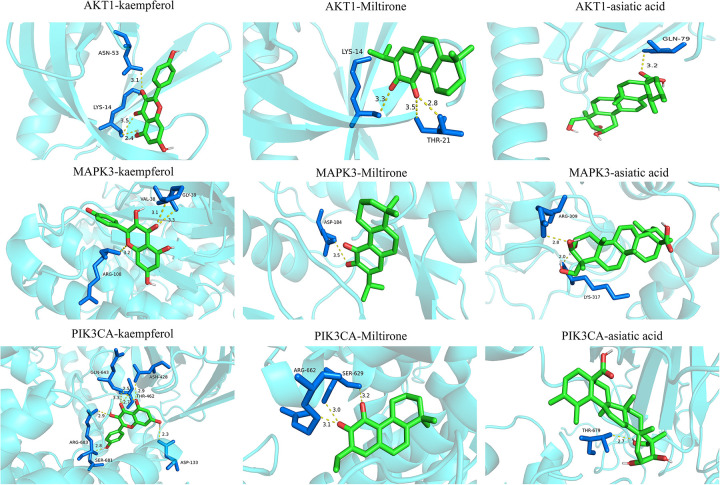
Binding models of key targets and ingredients.

Integrated screening via TCMSP, SwissTargetPrediction, and BATMAN-TCM identified 114 bioactive components and 1,614 potential targets of GXST (Table 2; Figure 4A). Meanwhile, 1,939 UA-related targets were retrieved from five disease databases (Figure 4B). A subsequent Venn analysis revealed 544 overlapping targets, representing the potential targets for GXST against UA **(**Figure 4C**)**. GO analysis indicated involvement in inflammatory response, MAPK cascade, and response to hypoxia (Supplementary Appendix 12). KEGG pathway analysis highlighted lipid metabolism and atherosclerosis, PI3K-Akt, and MAPK signaling (Figure 4D). Furthermore, PPI network construction and algorithmic ranking identified nine hub genes: TP53, EP300, AKT1, HRAS, HSP90AA1, MAPK1, MAPK3, PIK3CA, and PIK3CD (Figure 4E; Supplementary Appendix 13). Based on network degree values and KEGG results, top-ranked compounds kaempferol(degree = 132), miltirone(degree = 89), and asiatic acid (degree = 82) were selected for molecular docking with key targets(AKT1, MAPK3, PIK3CA). The docking results demonstrated strong binding affinities and stable conformations for all complexes, suggesting effective interactions between GXST compounds and UA targets (Table 3; Figure 5**)**.

**Table 2A:** Active components of guanxinshutong capsule. Active components of GXST identified from TCMSP.

English/Latin Name	Chinese Name	Molecule ID	Molecule Name	OB (%)	Caco-2	DL
*Choerospondias axillaris (Roxb.)* B.L.Burtt & A.W.Hill	Guangzao	MOL001040	(2R)-5,7-dihydroxy-2-(4-hydroxyphenyl) chroman-4-one	42.36	0.38	0.21
		MOL001490	bis[(2S)-2-ethylhexyl] benzene-1,2-dicarboxylate	43.59	0.98	0.68
		MOL001736	(-)-taxifolin	60.51	−0.24	0.27
		MOL000358	beta-sitosterol	36.91	1.32	0.75
		MOL000422	kaempferol	41.88	0.26	0.24
		MOL004328	naringenin	59.29	0.28	0.21
		MOL000096	(-)-catechin	49.68	−0.03	0.24
		MOL000098	quercetin	46.43	0.05	0.28
*Sal*via *miltiorrhiza* Bunge	Danshen	MOL001601	1,2,5,6-tetrahydrotanshinone	38.75	0.96	0.36
		MOL001659	Poriferasterol	43.83	1.44	0.76
		MOL001771	poriferast-5-en-3beta-ol	36.91	1.45	0.75
		MOL001942	isoimperatorin	45.46	0.97	0.23
		MOL002222	sugiol	36.11	1.14	0.28
		MOL002651	Dehydrotanshinone II A	43.76	1.02	0.4
		MOL000006	luteolin	36.16	0.19	0.25
		MOL006824	*α*-amyrin	39.51	1.37	0.76
		MOL007036	5,6-dihydroxy-7-isopropyl-1,1-dimethyl-2,3-dihydrophenanthren-4-one	33.77	1.19	0.29
		MOL007041	2-isopropyl-8-methylphenanthrene-3,4-dione	40.86	1.23	0.23
		MOL007045	3α-hydroxytanshinoneⅡa	44.93	0.53	0.44
		MOL007048	(E)-3-[2-(3,4-dihydroxyphenyl)-7-hydroxy-benzofuran-4-yl] acrylic acid	48.24	0.18	0.31
		MOL007049	4-methylenemiltirone	34.35	1.25	0.23
		MOL007050	2-(4-hydroxy-3-methoxyphenyl)-5-(3-hydroxypropyl)-7-methoxy-3-benzofurancarboxaldehyde	62.78	0.35	0.4
		MOL007058	formyltanshinone	73.44	0.54	0.42
		MOL007059	3-beta-Hydroxymethyllenetanshiquinone	32.16	0.38	0.41
		MOL007061	Methylenetanshinquinone	37.07	1.03	0.36
		MOL007063	przewalskin a	37.11	−0.26	0.65
		MOL007064	przewalskin b	110.32	0.34	0.44
		MOL007068	Przewaquinone B	62.24	0.39	0.41
		MOL007069	przewaquinone c	55.74	0.42	0.4
		MOL007070	(6S,7R)-6,7-dihydroxy-1,6-dimethyl-8,9-dihydro-7H-naphtho[8,7-g] benzofuran-10,11-dione	41.31	−0.06	0.45
		MOL007071	przewaquinone f	40.31	−0.09	0.46
		MOL007077	sclareol	43.67	0.84	0.21
		MOL007079	tanshinaldehyde	52.47	0.57	0.45
		MOL007081	Danshenol B	57.95	0.53	0.56
		MOL007082	Danshenol A	56.97	0.33	0.52
		MOL007085	Salvilenone	30.38	1.46	0.38
		MOL007088	cryptotanshinone	52.34	0.95	0.4
		MOL007093	dan-shexinkum d	38.88	0.67	0.55
		MOL007094	danshenspiroketallactone	50.43	0.88	0.31
		MOL007098	deoxyneocryptotanshinone	49.4	0.85	0.29
		MOL007100	dihydrotanshinlactone	38.68	1.26	0.32
		MOL007101	dihydrotanshinoneⅠ	45.04	0.95	0.36
		MOL007105	epidanshenspiroketallactone	68.27	0.9	0.31
		MOL007107	C09092	36.07	1.63	0.25
		MOL007108	isocryptotanshi-none	54.98	0.93	0.39
		MOL007111	Isotanshinone II	49.92	1.03	0.4
		MOL007115	manool	45.04	1.28	0.2
		MOL007118	microstegiol	39.61	1.05	0.28
		MOL007119	miltionone Ⅰ	49.68	0.35	0.32
		MOL007120	miltionone Ⅱ	71.03	0.62	0.44
		MOL007121	miltipolone	36.56	0.5	0.37
		MOL007122	Miltirone	38.76	1.23	0.25
		MOL007123	miltirone Ⅱ	44.95	0.04	0.24
		MOL007124	neocryptotanshinone ii	39.46	0.76	0.23
		MOL007125	neocryptotanshinone	52.49	0.35	0.32
		MOL007127	1-methyl-8,9-dihydro-7H-naphtho[5,6-g] benzofuran-6,10,11-trione	34.72	0.5	0.37
		MOL007130	prolithospermic acid	64.37	0.1	0.31
		MOL007132	(2R)-3-(3,4-dihydroxyphenyl)-2-[(Z)-3-(3,4-dihydroxyphenyl) acryloyl] oxy-propionic acid	109.38	−0.33	0.35
		MOL007140	(Z)-3-[2-[(E)-2-(3,4-dihydroxyphenyl) vinyl]-3,4-dihydroxy-phenyl] acrylic acid	88.54	−0.09	0.26
		MOL007141	salvianolic acid g	45.56	−0.14	0.61
		MOL007143	salvilenone Ⅰ	32.43	1.13	0.23
		MOL007145	salviolone	31.72	1.04	0.24
		MOL007149	NSC 122421	34.49	1.08	0.28
		MOL007150	(6S)-6-hydroxy-1-methyl-6-methylol-8,9-dihydro-7H-naphtho[8,7-g] benzofuran-10,11-quinone	75.39	0.03	0.46
		MOL007151	Tanshindiol B	42.67	0.05	0.45
		MOL007152	Przewaquinone E	42.85	−0.04	0.45
		MOL007154	tanshinone iia	49.89	1.05	0.4
		MOL007155	(6S)-6-(hydroxymethyl)-1,6-dimethyl-8,9-dihydro-7H-naphtho[8,7-g] benzofuran-10,11-dione	65.26	0.44	0.45
		MOL007156	tanshinone Ⅵ	45.64	0.48	0.3
*Syzygium aromaticum (L.)* Merr. & L.M.Perry	Dingxiang	MOL013219	Strictosamide_qt	76.3	0.59	0.76
		MOL001749	ZINC03860434	43.59	1.04	0.35
		MOL000358	beta-sitosterol	36.91	1.32	0.75
		MOL000422	kaempferol	41.88	0.26	0.24
		MOL000449	Stigmasterol	43.83	1.44	0.76
		MOL000098	quercetin	46.43	0.05	0.28
*Blumea balsamifera (L.)* DC	Bingpian	MOL006861	asiatic acid	41.38	−0.29	0.71
		MOL006862	bronyl acetate	59.3	0.23	0.51
		MOL006865	dipterocarpol	41.71	1.01	0.76
*Bambusa textilis* McClure	Tianzhuhuang	Not Found	Not Found	Not Found	Not Found	Not Found

Represents the chemical components of five botanical drugs in GXST, which were retrieved from TCMSP (http://tcmspw.com/tcmsp.php), the world's largest non-commercial TCM database. To ensure comprehensive target identification, BATMAN-TCM (http://bionet.ncpsb.org.cn/batman-tcm/index.php) was also employed, with results summarized in Table 2B. Collectively, 114 pharmacologically active constituents were identified to evaluate the efficacy of GXST in treating UA.

**Table 2B:** Active components of GXST identified from BATMAN-TCM.

English/Latin Name	Chinese Name	Compounds
Choerospondias axillaris (Roxb.) B.L.Burtt & A.W.Hill	Guangzao	Camp, Glucose, Jujuboside B, Cgmp
*Salvia miltiorrhiza Bunge*	Danshen	Tanshiquinone B, Miltirone, Isocucurbitacin D, Monomethyl Lithospermate, Salonitenolide, Magnesium Lithospermate B, Miltionone I, Lithospermic Acid, Neotanshinone C, Salvinone, Cryptoxanthin, DanshenxinkunA,Dehydromiltirone,Ferruginol,Neocryptotanshinone,Danshensu,Isocryptotanshinone,Caffeicacid,Neocryptotanshinone Ii, Heteratisine, Isotenulin, Dihydroisotanshinone I, Tanshinone Vi, Salvianolicacid B,Daphneolone, Tanshinone Ii A, Dehydrotremetone, Gamma-Sitosterol, Lithospermate B, Dauricine, Rosmarinine, 6-Hydroxymethyllumazin, 7-Hydroxymethyllumazin, Ta-nshinone Iib, Samaderin A, 1-Hydroxytaxinine A, 2-Hydroxytaxinine A, 3-Hydroxytaxinine A, 4-Hydroxytaxinine A, Isotanshinone Ii,Ursolicacid, Dihydrokaranone, Salviol
*Syzygium aromaticum (L.)* Merr. & L.M.Perry	Dingxiang	Methyl Salicylate, Kaempferol, Isocembrol, Methyl Phenylacetate, Beta-Humulene, Eugenone, Eugenitin, Oleanolic Acid-28-O-Beta-D-Glucopyranoside, Isoeugenol, Alpha-Humulene, Oleanolic Acid,3'-O-Methyl Sappanol, Eugenol, Isoeruboside B, Chavicol, Benzyl Acetate, Eugenin, Alpha-Ylangene
*Blumea balsamifera (L.)* DC	Bingpian	Borneol, D-Borneol, Erythrodiol, Dipterocarpol, Asiatic Acid, Dryocrassin, Camphor,Elemicin, Dryobalanone, (+)-Erythro-Guaiacylglycerol, Alphitolic Acid
*Bambusa textilis* McClure	Tianzhuhuang	Choline, Lycine

**Table 3:** Binding affinities of molecular docking.

Compounds	kaempferol	Miltirone	asiatic acid
Protein	Binding affinity (kcal/mol)	Binding affinity (kcal/mol)	Binding affinity (kcal/mol)
AKT1	−6.7	−6.6	−7.0
MAPK3	−7.5	−7.4	−8.0
PIK3CA	−8.5	−8.6	−9.2

## 4 Discussion

This meta-analysis systematically evaluated the effectiveness and safety of GXST combined with WM against WM monotherapy in patients with UA. All 15 RCTs included in this study strictly adhered to the efficacy evaluation standards for UA specified in the GCRNCM, ensuring standardized outcome consistency in defining, measuring, and calculating, effectively mitigating potential confounding from assessment variability in the pooled analysis. The combination therapy significantly elevated the clinical effectiveness rate and ECG efficacy rate, while reducing TCM syndrome scores, PV and FIB levels, microcirculatory disturbance, and improving angina symptoms. However, no significant intergroup differences were observed in CK-MB, cTnI, or ADRI. Although Begg's test and funnel plots suggested potential publication bias, sensitivity analysis and TSA confirmed the robustness of the findings.

In terms of mechanisms, network pharmacology elucidated that the therapeutic effects of GXST are orchestrated through multi-target modulation of key pathways, notably lipid metabolism, atherosclerosis, and the PI3K-Akt and MAPK signaling cascades. As illustrated in Figure 4, bioinformatic analysis identified 114 bioactive components in GXST and 544 overlapping targets with UA. Core components such as kaempferol, miltirone, and asiatic acid were found to engage key targets, including AKT1, MAPK3, and PIK3CA, within the enriched PI3K-Akt and MAPK pathways (Figures 4D,E). These targets regulate pivotal UA-related processes such as platelet activation, cardiomyocyte apoptosis, and inflammatory response, which exactly explains the clinical effects of GXST combined with WM in improving angina symptoms and reducing PV and FIB levels in the meta-analysis, reflecting the synergistic therapeutic advantage of traditional Chinese medicine compounds with “multi-component, multi-target, and multi-pathway”.

Molecular docking further corroborated these interactions, demonstrating stable binding between the key bioactive compounds and the core targets. Clinically, the meta-analysis showed that GXST combined with WM significantly increased the clinical effective rate (RR = 1.19) and ECG effective rate (RR = 1.20), while reducing angina attacks, and improving hemorheological parameters. The network pharmacology findings offer a mechanistic bridge to these outcomes. For instance, kaempferol's engagement with AKT1 may enhance vascular endothelial function, corresponding to the observed decrease in plasma viscosity. Moreover, the holistic regulation exerted by GXST through concurrent modulation of AKT1 and MAPK3 via multiple components reflects the UA's core pathogenesis “blood stasis blocking collaterals” and TCM principle of “promoting blood circulation and removing stasis,” which is highly consistent with the mechanism revealed by network pharmacology—“regulating lipid metabolism, atherosclerosis, and the PI3K-Akt pathway to improve vascular endothelial function and inhibit thrombosis”. This concordance between TCM theory, pathway-based mechanism, and clinical outcomes strengthens the validity and translational relevance of the study conclusions.

In TCM, UA is classified as “chest impediment” or “true heart pain”, primarily characterized by “deficient yang and excessive yin”. Contemporary TCM scholars emphasize “deficiency”, “phlegm”, and “blood stasis” as crucial factors (62), with “heart vessel blockage stasis” constituting the core pathogenesis (63). “Phlegm-dampness” correlates with hyperlipidemia and atherosclerosis, while “blood stasis” reflects impaired hemorheology and myocardial ischemia (64). GXST, the first state-approved Mongolian medicine for CHD and angina pectoris (65), has demonstrated significant efficacy in treating UA. Its formulation combines *Choerospondias axillaris (Roxb.)* (monarch herb) and *Salvia miltiorrhiza* (minister herb) to promote qi and blood circulation, alleviate blood stasis, and regenerate blood. *Syzygium aromaticum (L.)* warms the middle-energizer and strengthens yang qi, *Blumea balsamifera (L.)* clears heat and relieves pain, and *Bambusa textilis* clears heat and resolves phlegm. Together, these components act synergistically, using both warming and clearing methods, to promote circulation, resolve stasis, regulate qi, and alleviate pain, thereby embodying a macro-regulatory strategy for UA.

Modern pharmacological studies have shown that GXST mitigates inflammation, stabilizes atherosclerotic plaques, inhibits platelet aggregation and thrombosis, and improves hemorheology (66, 67), thereby alleviating myocardial ischemia and angina. Moreover, GXST exerts protective effects against atherosclerosis by reducing lipid levels and protecting vascular endothelial cells, thereby suppressing its initiation and progression. Collectively, these multifactorial actions support its potential in the prevention and treatment of cardiovascular diseases (CVD) (36, 68).

Hemorheological alterations are critical pathological factors in UA that perpetuate the disease course (69). The primary etiology involves atherosclerotic plaque rupture, which is primarily attributable to lipid metabolism disorders and inflammatory cascades, along with impaired hemorheology (70). Following plaque rupture, dysregulated blood rheology promotes thrombosis and induces myocardial ischemia, ultimately triggering angina.

Several bioactive compounds in GXST exert protective effects against UA. Kaempferol protects the heart by upregulating the PI3K/AKT/Nrf2 pathway, thereby attenuating atherosclerosis, suppressing inflammation, and inhibiting cellular apoptosis (71). It also inhibits platelet activation via the MAPK and AKT pathways (72, 73). Miltirone, a potential anti-platelet component of *Salvia miltiorrhiza* (74), suppresses platelet aggregation and granule release by targeting the Syk-PLCγ2-PKC/MAPK and PI3K-Akt-GSK3β signaling. Asiatic acid (AA), an active constituent of *Blumea balsamifera*, modulates glycophagy- and mitophagy-mediated energy metabolism via PI3K/Akt and AMPK signaling pathways to protect ischemic cardiomyocytes (75). Additionally, Tanshinone IIA enhances plaque stability through TGF-*β*/PI3K/Akt/eNOS activation, while quercetin improves cardiomyocyte survival via AMPK/mTOR-regulated autophagy and apoptosis inhibition (76, 77).Phosphoinositide 3-kinases (PI3Ks) catalytic subunit p110α is encoded by PIK3CA (78). MAPK3, a mitogen-activated protein kinase (MAPK) family member, mediates the cardioprotective effects of cryptotanshinone by reducing myocardial apoptosis through enhanced activation (76). In summary, GXST may ameliorate UA by coordinately targeting the PIK3CA/AKT1 and MAPK3 signaling pathways. These multi-mechanistic effects underpin its Micro-targeted therapeutic approach in UA management.

Advancing age is a major risk factor for CHD, with an approximately 10-fold higher incidence in adults aged ≥75 years compared to those <55 years (79). Age-related structural changes in arterial walls compromise vascular compliance, elevate peripheral resistance, and increase cardiac workload, thereby exacerbating conditions such as hypertensive heart disease (80). Notably, hypertension and dyslipidemia are increasingly prevalent among younger populations, further amplifying cardiovascular risk. New-onset hypertriglyceridemia is associated with markedly elevated risks of CVD, particularly in individuals under 45 years (2.61-fold vs. controls) (81). Additionally, elevated blood pressure correlates positively with an increased risk of diabetes mellitus and prediabetes (82). Older adults are also more susceptible to nutritional deficiencies, which can trigger a cascade of adverse outcomes, including exacerbated comorbidities, accelerated disease progression, and an elevated risk of infectious complications, ultimately compromising overall prognosis and survival. Given the trend toward earlier onset of cardiovascular disorders, early detection and management of modifiable risk factors (hypertension, hyperglycemia, and hyperlipidemia) are crucial for preventing the development and progression of CHD.

Despite representing the most recent systematic review and meta-analysis in this field, several limitations must be acknowledged. First, the predominance of Chinese studies limits the generalizability of our findings. Future prospective studies involving diverse ethnic populations are essential to enhance external validity. Second, due to insufficient blinding and allocation concealment, the methodological quality of the included trials was limited, potentially introducing bias and compromising the robustness of the evidence. Third, the safety profile of GXST remains uncertain owing to inconsistent reporting of adverse events and a lack of long-term efficacy data. Although preliminary mechanistic insights have been elucidated, further experimental validation is necessary to confirm these findings. Therefore, our results should be interpreted with caution. Standardized and rigorously designed trials are crucial to generating high-quality evidence. Beyond establishing efficacy in large-scale RCTs, future research must integrate disease characteristics, physiological status, drug metabolism features, and dose-response relationships to personalize GXST therapy and optimize UA management.

## 5 Conclusion

In summary, as an adjunctive therapy, GXST significantly improves clinical efficacy in patients with UA by alleviating angina symptoms, optimizing hemodynamic parameters, and maintaining a favorable safety profile. Mechanistically, GXST may alleviate angina by regulating the PIK3CA/AKT1 and MAPK3 signaling pathways. Although these findings support the potential of GXST in UA management, further large-scale trials are warranted to validate its efficacy and explore long-term outcomes.

## Data Availability

The original contributions presented in the study are included in the article/Supplementary Material, further inquiries can be directed to the corresponding authors.
