# Supplementary material for: Efficacy and safety of guanxinshutong capsule as adjunctive therapy for unstable angina: an integrated systematic review, meta-analysis, and network pharmacology study
Source: Front Cardiovasc Med. 2026 Feb 24;13:1740334. doi: 10.3389/fcvm.2026.1740334 (PMC12971700; doi:10.3389/fcvm.2026.1740334)
Supplement: Supplementary file 2 [file Table2.docx]

**Appendix** **Outline**

**[Appendix 1：PRISMA checklist](#_Toc209050417)** [3](#_Toc209050417)

**[Appendix 2: Search strategy](#_Toc209050418)** [3](#_Toc209050418)

**[Table S1. Search strategy of PubMed](#_Toc209050419)** [3](#_Toc209050419)

**[Table S2. Search strategy of Embase](#_Toc209050420)** [4](#_Toc209050420)

**[Table S3. Search strategy of Cochrane Library](#_Toc209050421)** [5](#_Toc209050421)

**[Table S4.](#_Toc209050422)****[Search strategy of Web of Science](#_Toc209050422)** [5](#_Toc209050422)

**[Table S5. Search strategy of Chinese databases](#_Toc209050423)** [6](#_Toc209050423)

**[Appendix 3:](#_Toc209050424)****[Assessment of risk bias in randomized clinical trials](#_Toc209050424)** [6](#_Toc209050424)

**[Appendix 4: Labbé plots of effective clinical rate for heterogeneity](#_Toc209050425)** [7](#_Toc209050425)

**[Appendix 5: Funnel plots for PV、FIB、cTnI , and CK-MB](#_Toc209050426)** [8](#_Toc209050426)

**[Appendix 6: Funnel plots for publication bias](#_Toc209050427)** [8](#_Toc209050427)

**[Figure S6.1 Funnel plots of the effective clinical rate](#_Toc209050428)** [9](#_Toc209050428)

**[Figure S6.2 Funnel plots after conducting “trim - and - fill method”](#_Toc209050429)** [9](#_Toc209050429)

**[Appendix 7: The meta-regression for heterogeneity](#_Toc209050430)** [10](#_Toc209050430)

**[Appendix 8: The subgroup analyses for heterogeneity](#_Toc209050431)** [11](#_Toc209050431)

**[Figure S8.1 Subgroup analyses of the disease duration concerning the frequency of angina pectoris](#_Toc209050432)** [11](#_Toc209050432)

**[Figure S8.2 Subgroup analyses of the subject age concerning the frequency of angina pectoris](#_Toc209050433)** [11](#_Toc209050433)

**[Figure S8.3 Subgroup analyses of the drug treatment course concerning the frequency of angina pectoris](#_Toc209050434)** [12](#_Toc209050434)

**[Figure S8.4 Subgroup analyses of the disease duration concerning the duration of angina pectoris](#_Toc209050435)** [12](#_Toc209050435)

**[Figure S8.5 Subgroup analyses of the subject age concerning the duration of angina pectoris](#_Toc209050436)** [13](#_Toc209050436)

**[Figure S8.6 Subgroup analyses of the drug treatment course concerning the duration of angina pectoris](#_Toc209050437)** [13](#_Toc209050437)

**[Appendix 9: Sensitivity analysis of different outcome indicators](#_Toc209050438)** [14](#_Toc209050438)

**[Figure S9.1 Sensitivity analysis of the frequency of angina pectoris](#_Toc209050439)** [14](#_Toc209050439)

**[Figure S9.2 Sensitivity analysis of the duration of angina pectoris](#_Toc209050440)** [14](#_Toc209050440)

**[Figure S9.3 Forest plot after removing the study with the lowest weight](#_Toc209050441)** [15](#_Toc209050441)

**[Appendix 10.](#_Toc209050442)****[Grade evidence quality assessment for meta-analysis.](#_Toc209050442)** [16](#_Toc209050442)

**[Appendix 11: Trial Sequential Analysis](#_Toc209050443)** [17](#_Toc209050443)

**[Figure S11.1 Trial Sequential Analysis of the clinical effective rate](#_Toc209050444)** [17](#_Toc209050444)

**[Figure S11.2 Trial Sequential Analysis of the effective rate in ECG](#_Toc209050445)** [18](#_Toc209050445)

**[Figure S11.3 Trial Sequential Analysis of the frequency of angina pectoris](#_Toc209050446)** [19](#_Toc209050446)

**[Figure S11.4 Trial Sequential Analysis of the duration of angina pectoris](#_Toc209050447)** [20](#_Toc209050447)

**[Figure S11.5 Trial Sequential Analysis of the adverse drug reactions incidence](#_Toc209050448)** [21](#_Toc209050448)

**[Appendix 12: GO enrichment analyses of drug-disease targets.](#_Toc209050449)** [22](#_Toc209050449)

**[Appendix 13: Top 10 hub genes in six algorithms.](#_Toc209050450)** [23](#_Toc209050450)

**Appendix 1：****PRISMA checklist**

**Appendix 2:** **Search strategy**

**Table S1. Search strategy of PubMed**

| # | **searches** | results |  |
| --- | --- | --- | --- |
| 1 | "Angina,Unstable"[Mesh] | 11423 | |
| 2 | (((((((((((((((((("Angina, Unstable"[Mesh]) OR (Anginas, Unstable[Title/Abstract])) OR (Unstable Anginas[Title/Abstract])) OR (Angina at Rest[Title/Abstract])) OR (Angina, Preinfarction[Title/Abstract])) OR (Anginas, Preinfarction[Title/Abstract])) OR (Preinfarction Angina[Title/Abstract])) OR (Preinfarction Anginas[Title/Abstract])) OR (Unstable Angina[Title/Abstract])) OR (Angina Pectoris, Unstable[Title/Abstract])) OR (Angina Pectori, Unstable[Title/Abstract])) OR (Unstable Angina Pectori[Title/Abstract])) OR (Unstable Angina Pectoris[Title/Abstract])) OR (Myocardial Preinfarction Syndrome[Title/Abstract])) OR (Myocardial Preinfarction Syndromes[Title/Abstract])) OR (Preinfarction Syndrome, Myocardial[Title/Abstract])) OR (Preinfarction Syndromes, Myocardial[Title/Abstract])) OR (Syndrome, Myocardial Preinfarction[Title/Abstract])) OR (Syndromes, Myocardial Preinfarction[Title/Abstract]) | 21180 | |
| 3 | "guanxin shutong" [Supplementary Concept] | 10 | |
| 4 | ("guanxin shutong" [Supplementary Concept]) OR (guanxin shutong[Title/Abstract]) | 16 | |
| 5 | ((((((((((((((((((("Angina, Unstable"[Mesh]) OR (Anginas, Unstable[Title/Abstract])) OR (Unstable Anginas[Title/Abstract])) OR (Angina at Rest[Title/Abstract])) OR (Angina, Preinfarction[Title/Abstract])) OR (Anginas, Preinfarction[Title/Abstract])) OR (Preinfarction Angina[Title/Abstract])) OR (Preinfarction Anginas[Title/Abstract])) OR (Unstable Angina[Title/Abstract])) OR (Angina Pectoris, Unstable[Title/Abstract])) OR (Angina Pectori, Unstable[Title/Abstract])) OR (Unstable Angina Pectori[Title/Abstract])) OR (Unstable Angina Pectoris[Title/Abstract])) OR (Myocardial Preinfarction Syndrome[Title/Abstract])) OR (Myocardial Preinfarction Syndromes[Title/Abstract])) OR (Preinfarction Syndrome, Myocardial[Title/Abstract])) OR (Preinfarction Syndromes, Myocardial[Title/Abstract])) OR (Syndrome, Myocardial Preinfarction[Title/Abstract])) OR (Syndromes, Myocardial Preinfarction[Title/Abstract])) AND (("guanxin shutong" [Supplementary Concept]) OR (guanxin shutong[Title/Abstract])) | 0 | |

**Table S2. Search strategy of Embase**

| # | **searches** | results |
| --- | --- | --- |
| 1 | 'unstable angina pectoris'/exp | 28970 |
| 2 | 'anginas, unstable':ab,ti OR 'unstable anginas':ab,ti OR 'angina at rest':ab,ti OR 'angina, preinfarction':ab,ti OR 'anginas, preinfarction':ab,ti OR 'preinfarction angina':ab,ti OR 'preinfarction anginas':ab,ti OR 'unstable angina':ab,ti OR 'angina pectoris, unstable':ab,ti OR 'angina pectori, unstable':ab,ti OR 'unstable angina pectoris':ab,ti OR 'myocardial preinfarction syndrome':ab,ti OR 'myocardial preinfarction syndromes':ab,ti OR 'preinfarction syndrome, myocardial':ab,ti OR 'preinfarction syndromes, myocardial':ab,ti OR 'syndrome, myocardial preinfarction':ab,ti OR 'syndromes, myocardial preinfarction':ab,ti | 23036 |
| 3 | #1 OR #2 | 34986 |
| 4 | 'guanxin shutong'/exp | 10 |
| 5 | guanxinshutong:ab,ti | 16 |
| 6 | #4 OR #5 | 18 |
| 7 | #3 AND #6 | 0 |

**Table S3. Search strategy of Cochrane Library**

| # | **searches** | results |
| --- | --- | --- |
| 1 | MeSH descriptor: [Angina, Unstable] explode all trees | 1461 |
| 2 | (Anginas, Unstable or Unstable Anginas or Angina at Rest or Angina, Preinfarction or Anginas, Preinfarction or Preinfarction Angina or Preinfarction Anginas or Unstable Angina or Angina Pectoris, Unstable or Angina Pectori, Unstable or Unstable Angina Pectori or Unstable Angina Pectoris or Myocardial Preinfarction Syndrome or Myocardial Preinfarction Syndromes or Preinfarction Syndrome, Myocardial or Preinfarction Syndromes, Myocardial or Syndrome, Myocardial Preinfarction or Syndromes, Myocardial PreinfarctionP):ti,ab,kw | 6145 |
| 3 | #1 OR #2 | 6218 |
| 4 | (guanxin shutong):ti,ab,kw | 4 |
| 5 | #3 AND #4 | 0 |

**Table S4.** **Search strategy of Web of Science**

| # | **searches** |  |
| --- | --- | --- |
| 1 | Anginas, Unstable (Topic) or Unstable Anginas (Topic) or Angina at Rest (Topic) or Angina, Preinfarction (Topic) or Anginas, Preinfarction (Topic) or Preinfarction Angina (Topic) or Preinfarction Anginas (Topic) or Unstable Angina (Topic) or Angina Pectoris, Unstable (Topic) or Angina Pectori, Unstable (Topic) or Unstable Angina Pectori (Topic) or Unstable Angina Pectoris (Topic) or Myocardial Preinfarction Syndrome (Topic) or Myocardial Preinfarction Syndromes (Topic) or Preinfarction Syndrome, Myocardial (Topic) or Preinfarction Syndromes, Myocardial (Topic) or Syndrome, Myocardial Preinfarction (Topic) or Syndromes, Myocardial Preinfarction (Topic) | 34698 |
| 2 | Guanxin shutong capsule (Topic) or guanxin shutong (Topic) | 9 |
| 3 | #1 AND #2 | 1 |

**Table S5. Search strategy of Chinese databases**

| databases | **searches** | results |
| --- | --- | --- |
| CNKI | (theme: unstable angina pectoris) OR (abstract: 'unstable angina pectoris' OR 'angina pectoris' OR 'coronary heart disease (CHD)' OR 'myocardial infarction' OR 'acute coronary syndrome (accurately)) AND (theme: guanxin shutong capsule) OR (abstract:' guanxin shutong capsule 'OR' guanxin shutong '(accurately)) | 156 |
| Wanfang | (Theme: Guanxin Shutong Capsule) OR (title or keyword: (Guanxin Shutong capsule or Guanxin Shutong)) and (Theme: (Unstable Angina pectoris) OR title or keyword: Unstable Angina pectoris or coronary heart disease or angina pectoris or myocardial infarction or acute coronary syndrome)) | 250 |
| VIP | (((((Title or keyword = Unstable angina pectoris) OR Title or keyword = myocardial infarction) OR Title or keyword = Coronary heart disease) OR Title or keyword = Acute coronary syndrome) AND (Title or keyword = Guanxinshutong capsule OR Title or keyword = Guanxinshutong)) | 125 |
| SinoMed | (" Guanxin Shutong Capsule "[common field: intelligent] OR "Guanxin Shutong "[common field: intelligent]) AND (" Unstable angina pectoris "[common field: intelligent] OR" Angina pectoris "[common field: intelligent] OR "Coronary heart disease "[common field: intelligent] OR" Myocardial infarction "[common field: intelligent] OR "Acute coronary Syndrome "[Common field: intelligent]) | 133 |

**Appendix 3:** **Assessment of risk bias in randomized clinical trials**

| Study ID | Randomization  process | Deviations from intended interventions | Mising  outcome data | Measurement of the outcome | Selection of the reported result | Over all |
| --- | --- | --- | --- | --- | --- | --- |
| HUANG P(2023) | Low | Some concerns | Low | High | High | High |
| QIN Y(2022) | High | Some concerns | Low | High | Some concerns | High |
| GOU W(2020) | Low | Some concerns | Low | High | Some concerns | High |
| SONG S(2020) | High | Some concerns | Low | High | Some concerns | High |
| ZHAO J(2020) | Some concerns | Some concerns | Low | High | Some concerns | High |
| DU Y(2019) | High | Some concerns | Low | High | Some concerns | High |
| CHEN C(2018) | High | Some concerns | Low | High | Some concerns | High |
| MIAO L(2017) | Some concerns | Some concerns | Low | High | Some concerns | High |
| LIN Y(2016) | Low | Some concerns | Low | High | Some concerns | High |
| CHEN Y(2016) | Low | Some concerns | Low | High | Some concerns | High |
| CHEN Y(2016) | Low | Some concerns | Low | High | Some concerns | High |
| ZHANG J(2015) | High | Some concerns | Low | High | Some concerns | High |
| ZHAO J(2015) | Some concerns | Some concerns | Low | High | Some concerns | High |
| LIANG B(2015) | Some concerns | Some concerns | Low | High | Some concerns | High |
| GUO D(2013) | Some concerns | Some concerns | Low | High | Some concerns | High |

**Appendix 4: Labbé plots of effective clinical rate for heterogeneity**


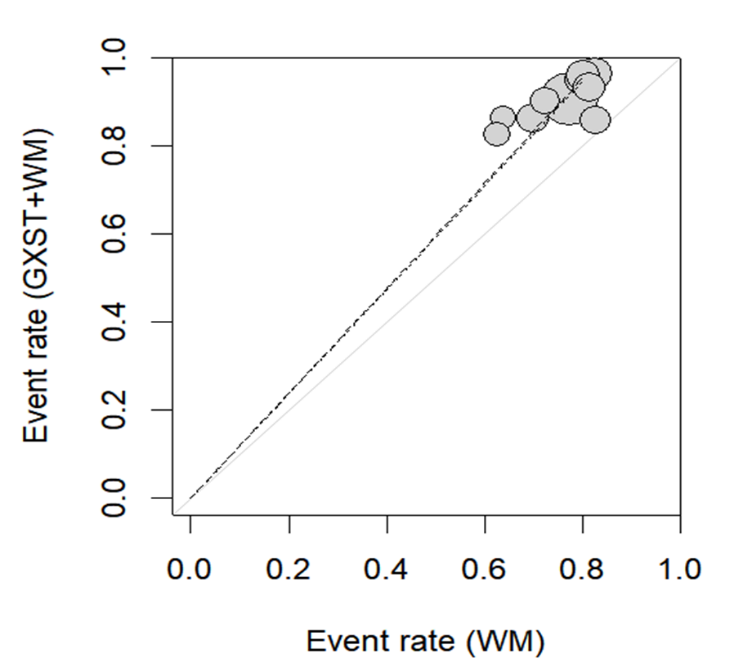


**Appendix 5: Funnel plots for PV、FIB、cTnI , and CK-MB**

**
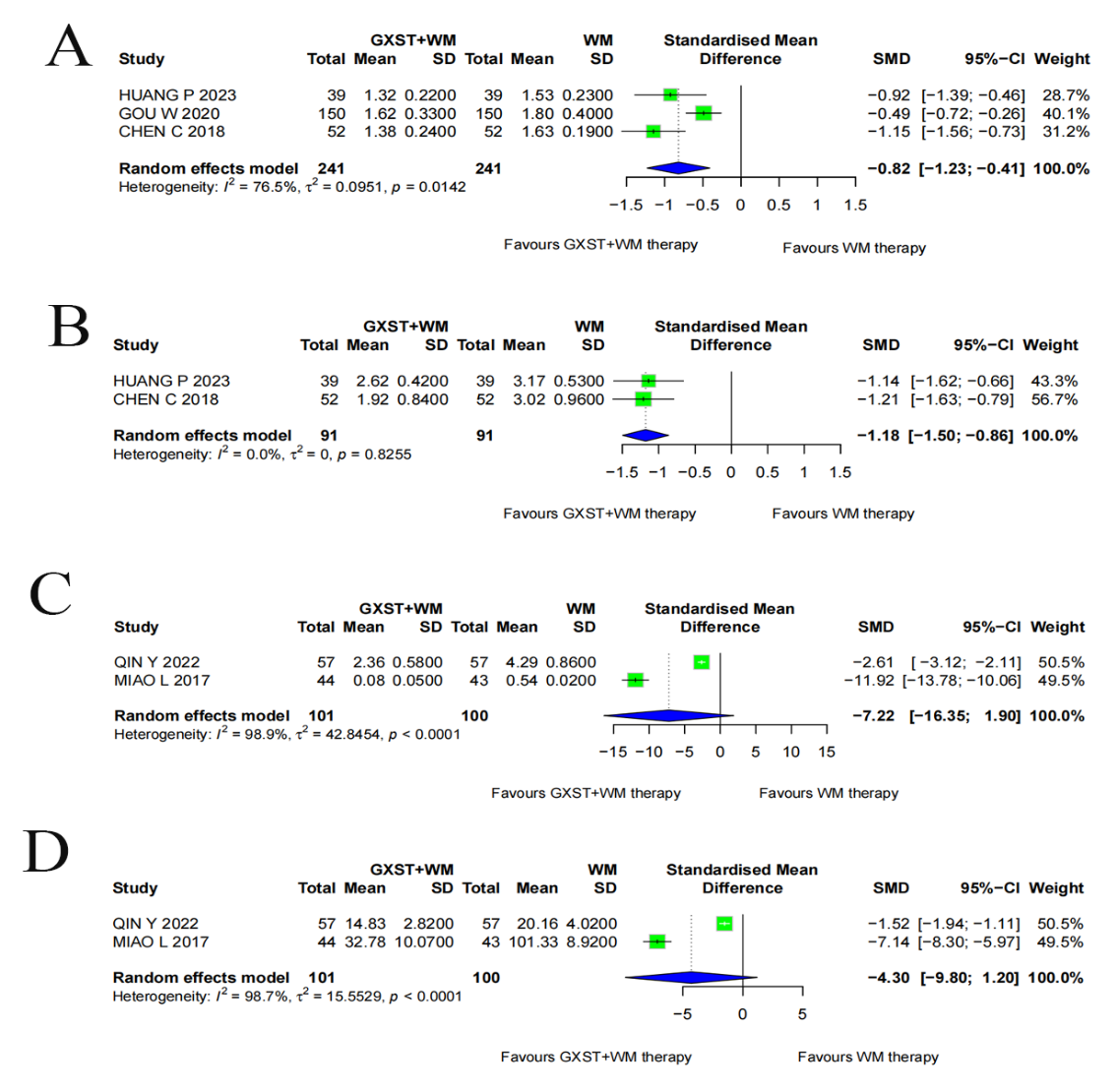
**

**(A)PV; (B)FIB; (C)cTnI; (D)CK-MB**

**Appendix 6: Funnel plots for publication bias**

**Figure S6.1 Funnel plots of the effective clinical rate**

**
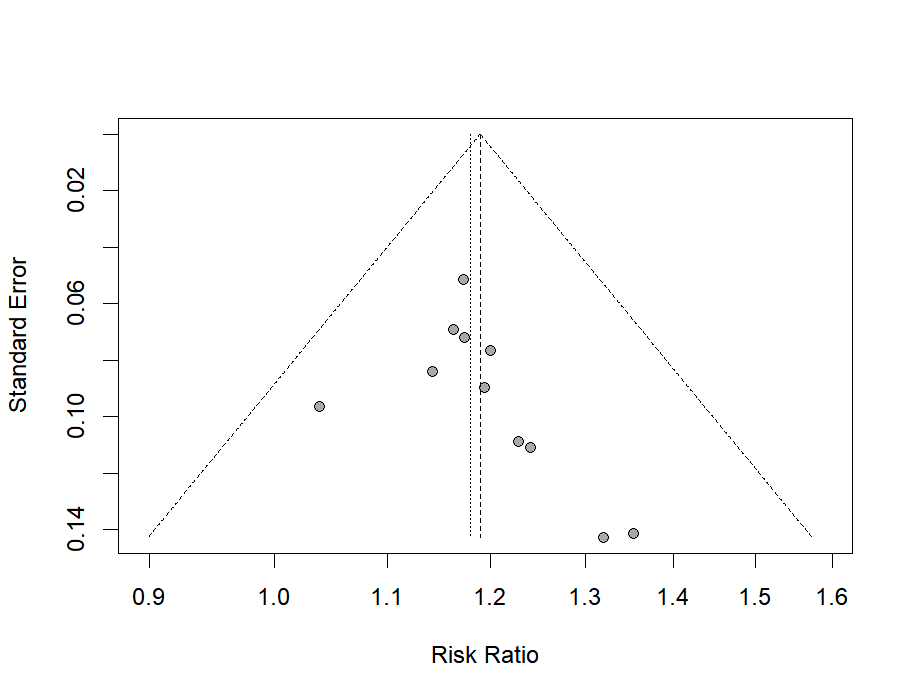
**

**Figure S6.2 Funnel plots after conducting “trim - and - fill method”**

**
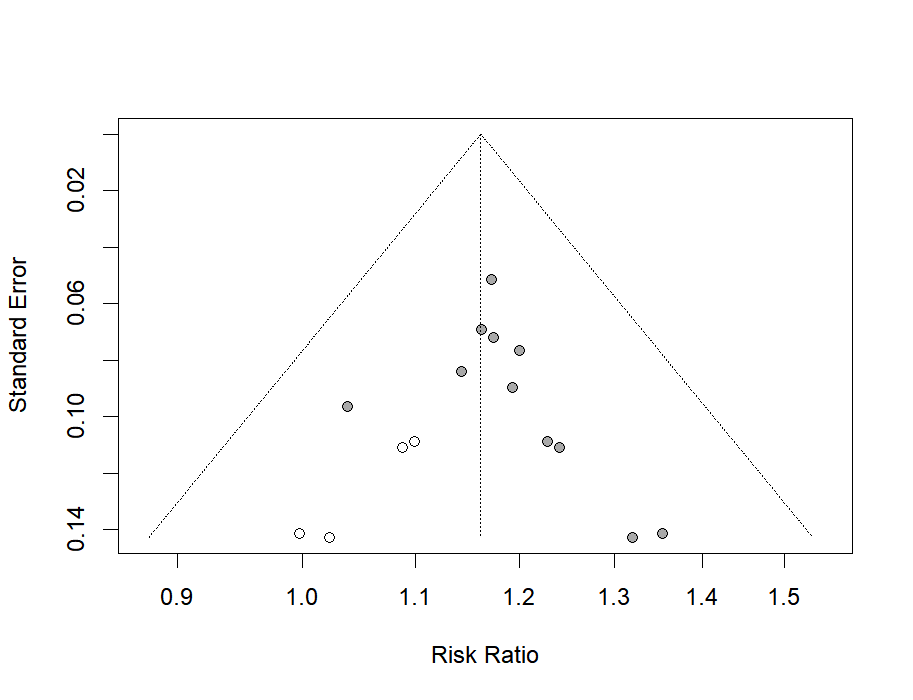
**

**Appendix 7: The meta-regression for heterogeneity**

| **Duration of angina pectoris**  **Frequency of angina pectoris** |  | **estimate** | **se** | **zval** | **pval** | **ci.lb** | **ci.ub** |
| --- | --- | --- | --- | --- | --- | --- | --- |
| **Duration of angina pectoris** | intrcpt | 1.1222 | 1.0771 | 1.0419 | 0.2974 | -0.9888 | 3.2332 |
|  | Time≤four weeks | -0.4926 | 0.7180 | -0.6861 | 0.4927 | -1.8999 | 0.9146 |
|  | Time ND | 1.6881 | 1.8104 | 0.9324 | 0.3511 | -1.8603 | 5.2364 |
|  | Age>55 years | -2.0660 | 1.0354 | -1.9954 | 0.0460 | -4.0953 | -0.0367 |
|  | Course>5 years | -1.9906 | 0.9525 | -2.0899 | 0.0366 | -3.8576 | -0.1237 |
|  | Course ND | -2.1613 | 1.4399 | -1.5010 | 0.1333 | -4.9834 | 0.6608 |
| **Frequency of angina pectoris** | intrcpt | 3.5257 | 0.6589 | 5.3512 | <.0001 | 2.2344 | 4.8171 |
|  | Time≤four weeks | -0.2642 | 0.3547 | -0.7450 | 0.4563 | -0.9594 | 0.4309 |
|  | Time ND | 6.4118 | 0.9707 | 6.6051 | <.0001 | 4.5092 | 8.3144 |
|  | Age>55 years | -5.0105 | 0.6401 | -7.8272 | <.0001 | -6.2652 | -3.7559 |
|  | Course>5 years | -4.2638 | 0.6144 | -6.9403 | <.0001 | -5.4680 | -3.0597 |
|  | Course ND | -6.6092 | 0.8021 | -8.2402 | <.0001 | -8.1812 | -5.0371 |

**Appendix 8: The** **subgroup analyses for** **heterogeneity**

**Figure S8.1 Subgroup analyses of the disease duration** **concerning the frequency of angina pectoris**

**
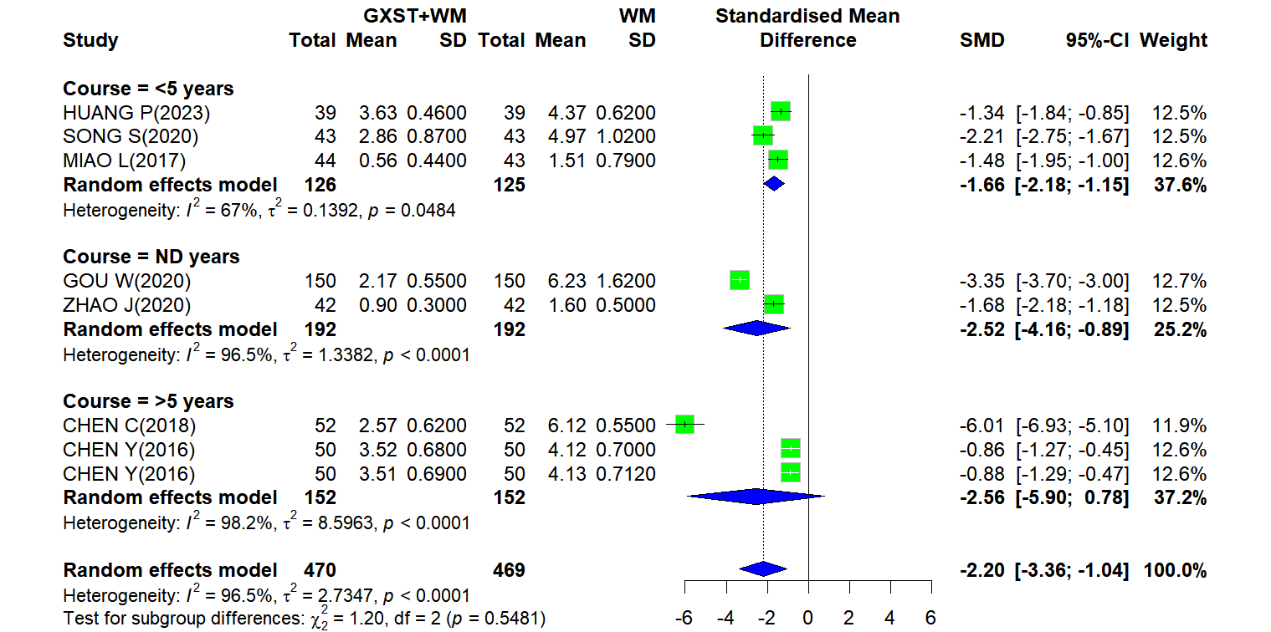
**

**Figure S8.2 Subgroup analyses of the subject age concerning the frequency of angina pectoris**

**
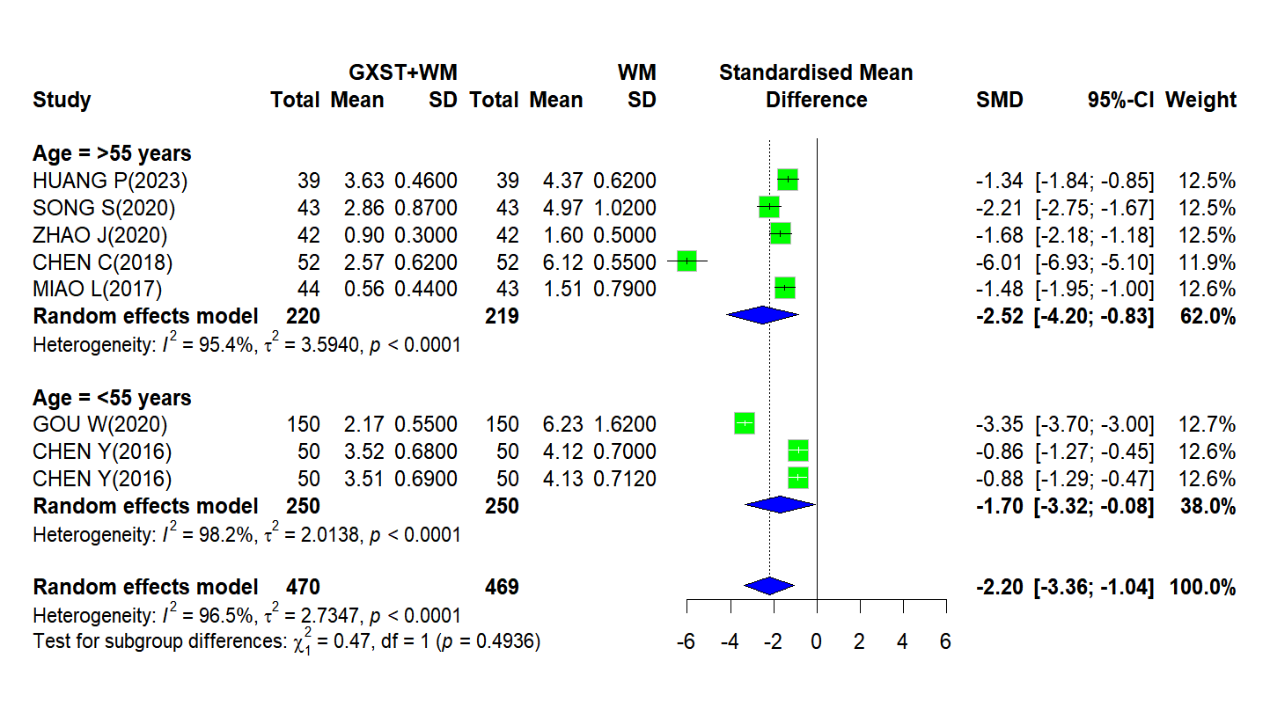
**

**Figure S8.3 Subgroup analyses of the drug treatment course concerning the frequency of angina pectoris**

**
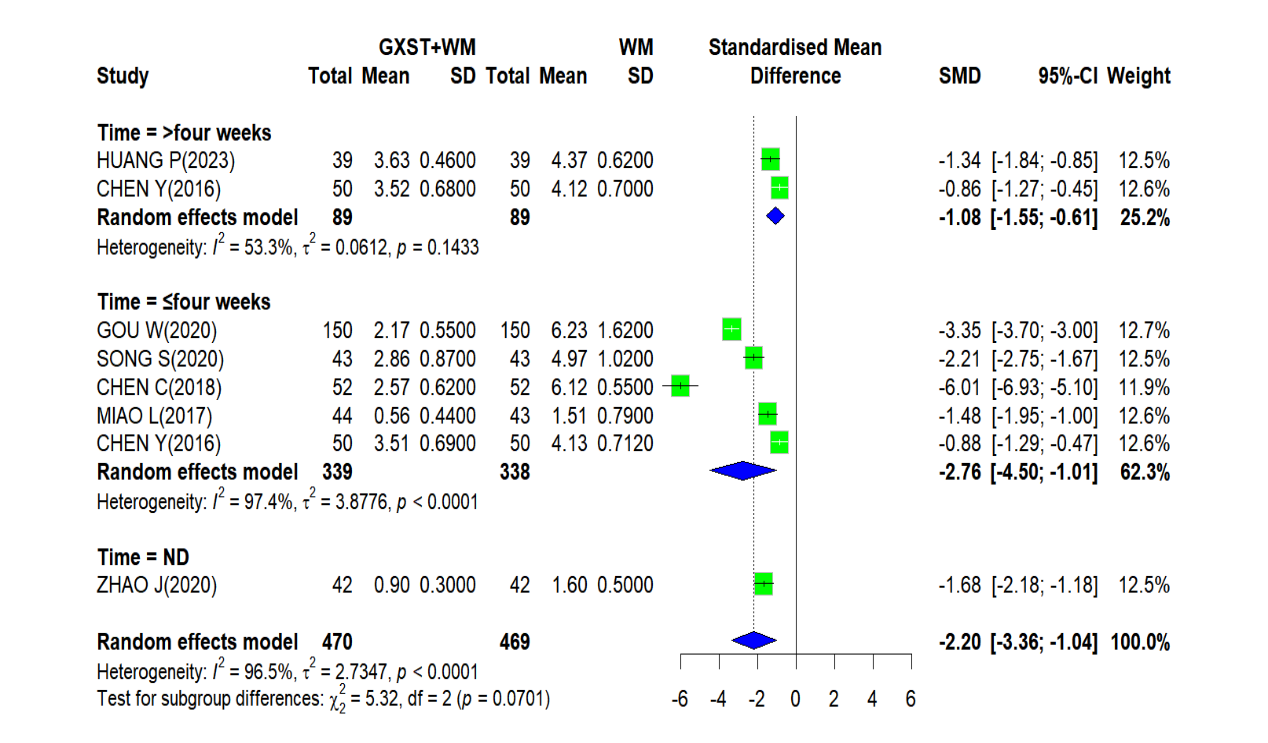
**

**Figure S8.4 Subgroup analyses of the disease duration concerning the duration of angina pectoris**

**
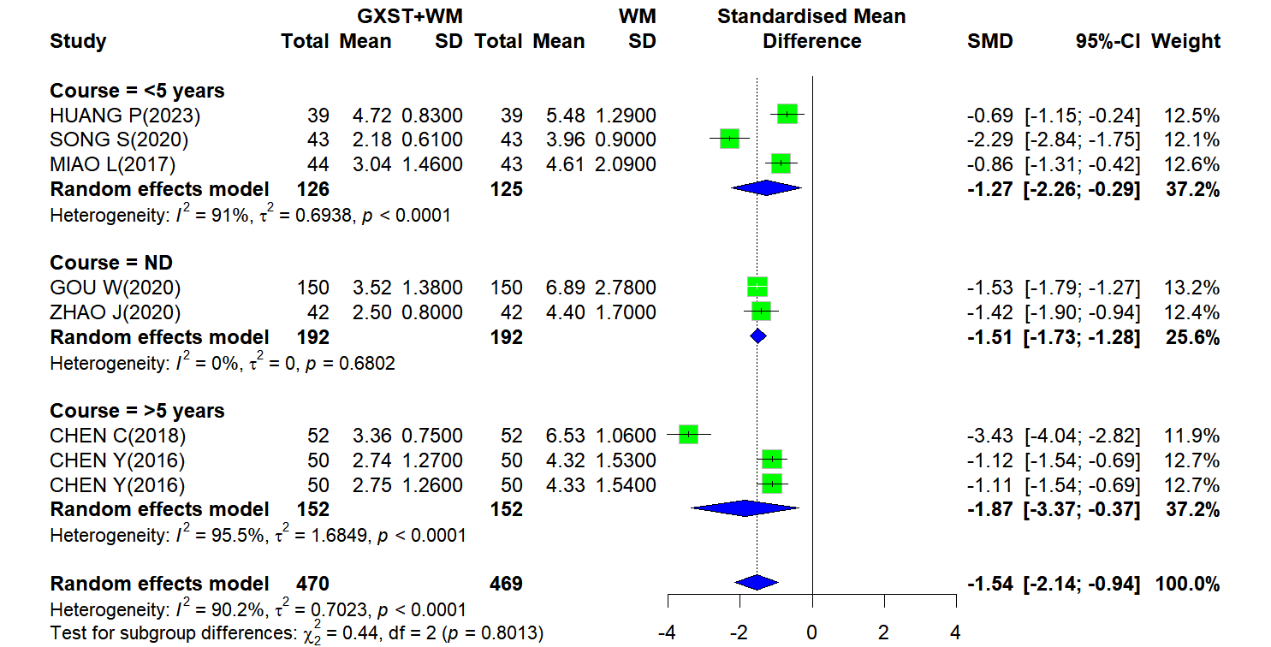
**

**Figure S8.5 Subgroup analyses of the subject age concerning** **the duration of angina pectoris**

**
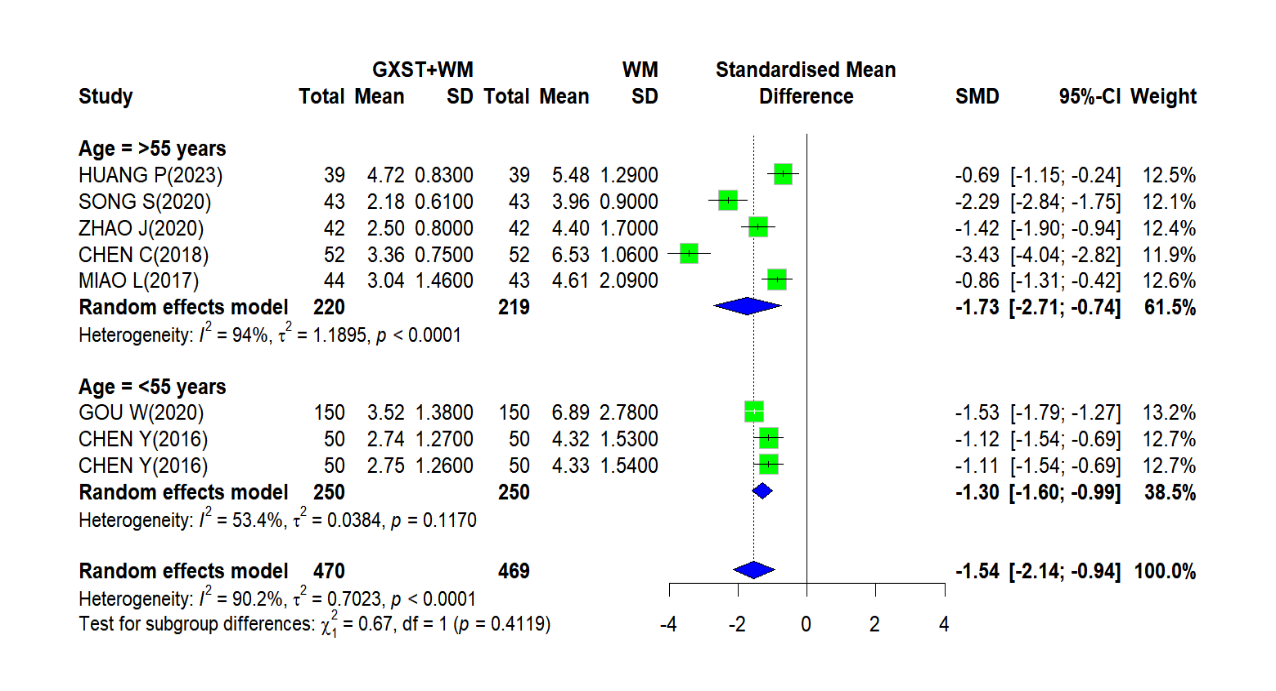
**

**Figure S8.6 Subgroup analyses of the drug treatment course concerning the duration of angina pectoris**

**
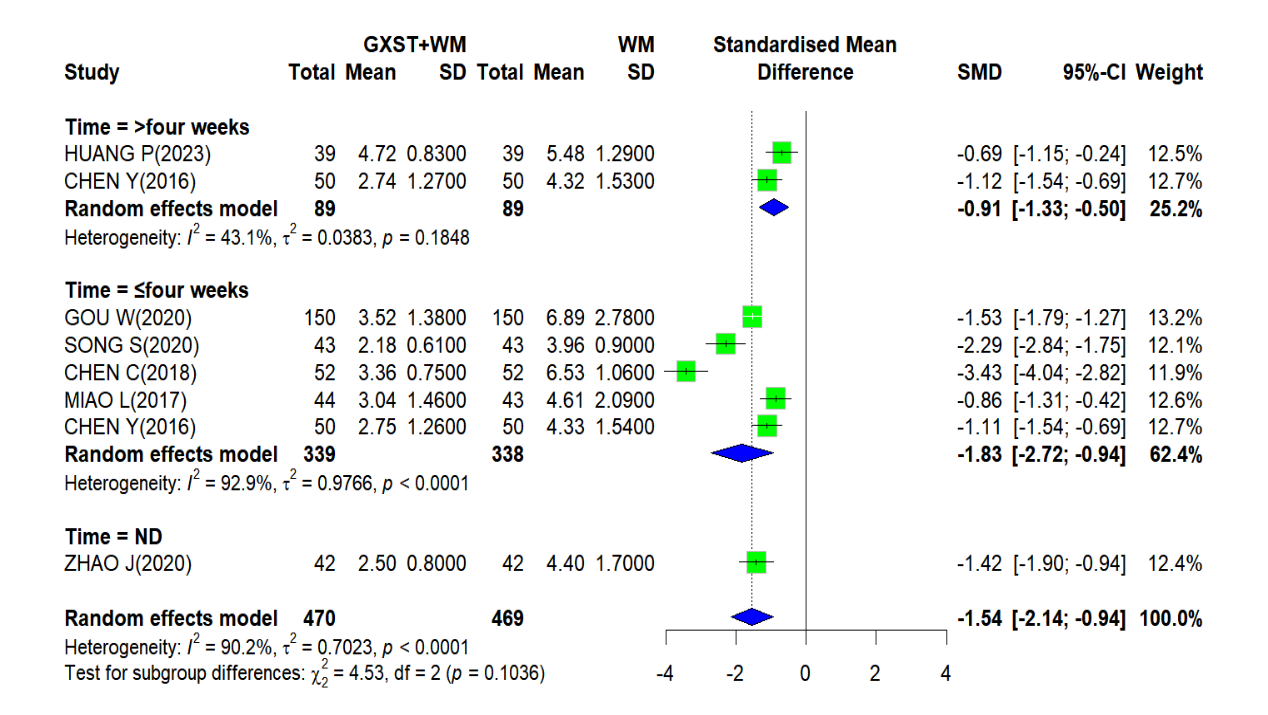
**

**Appendix 9:** **Sensitivity analysis of different outcome indicators**

**Figure S9.1 Sensitivity analysis of the frequency of angina pectoris**

**
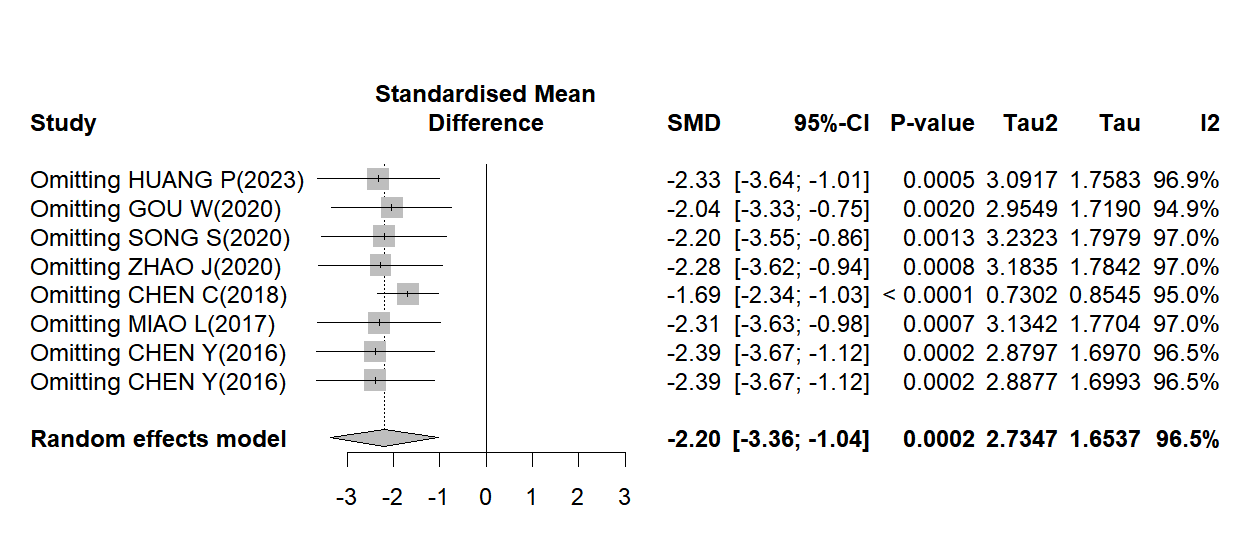
**

**Figure S9.2 Sensitivity analysis of the duration of angina pectoris**

**
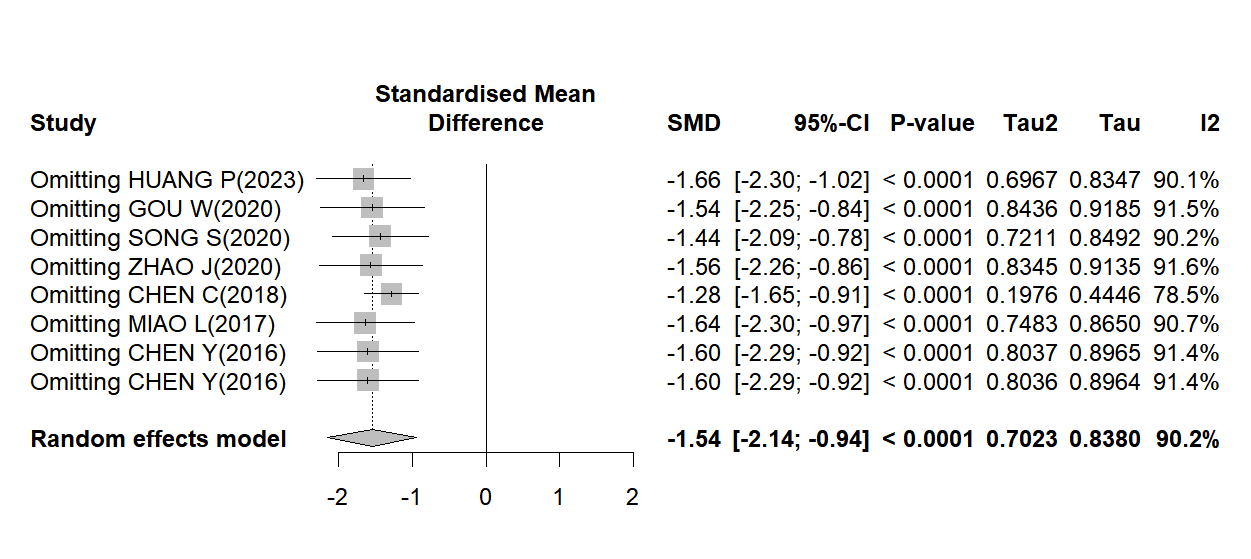
**

**Figure S9.3 Forest plot after removing the study with the lowest weight**

**
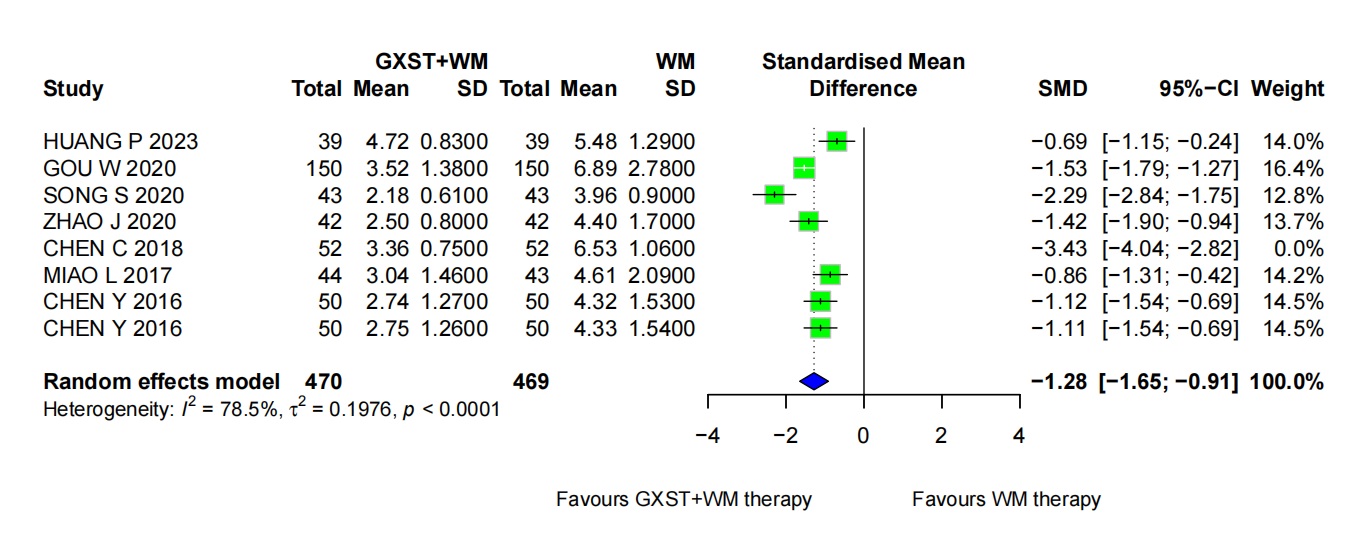
**

**Appendix 10.** **Grade evidence quality assessment for meta-analysis.**

**
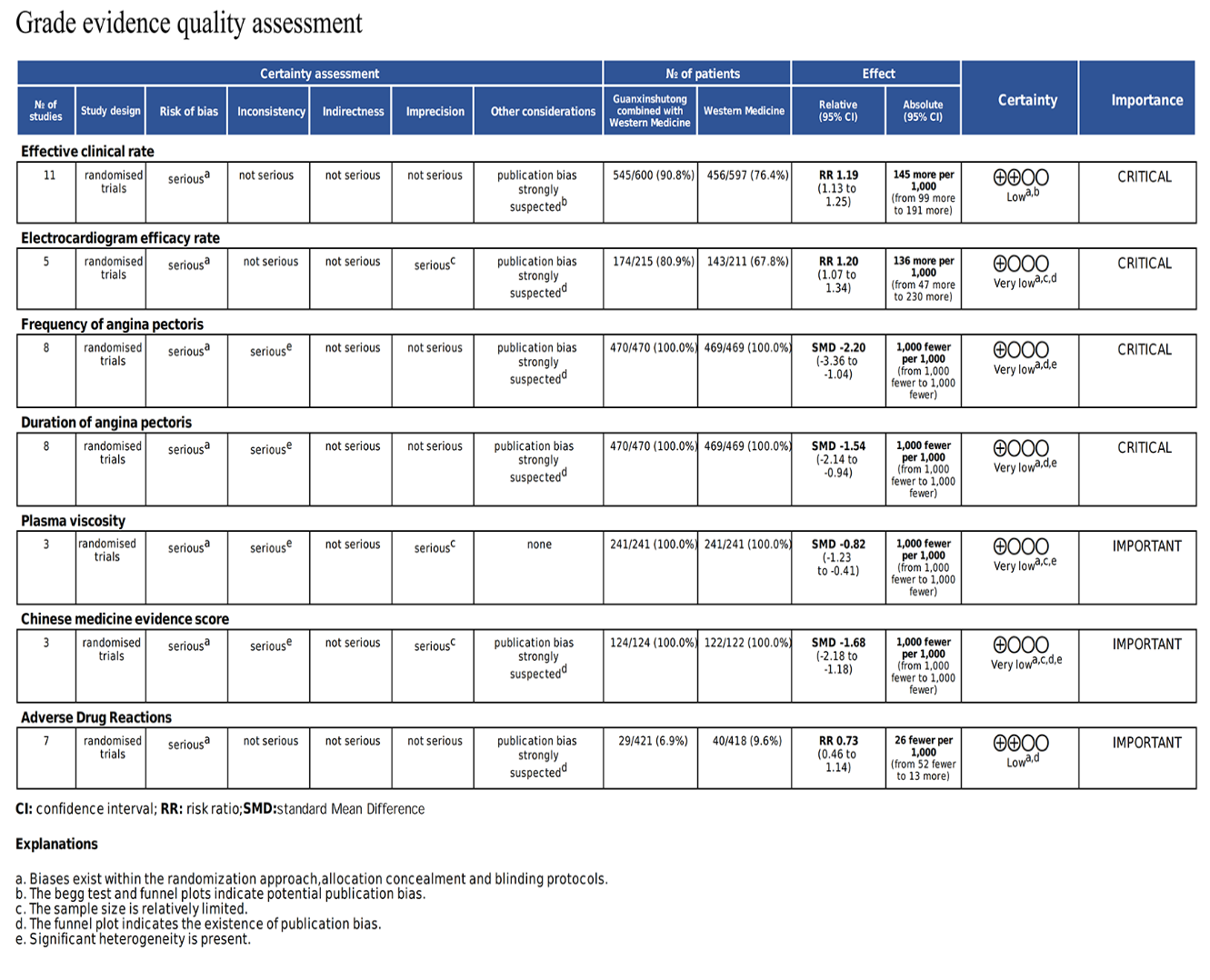
**

**Appendix 11: Trial Sequential Analysis**

**Figure S11.1 Trial Sequential Analysis of the clinical effective rate**

**
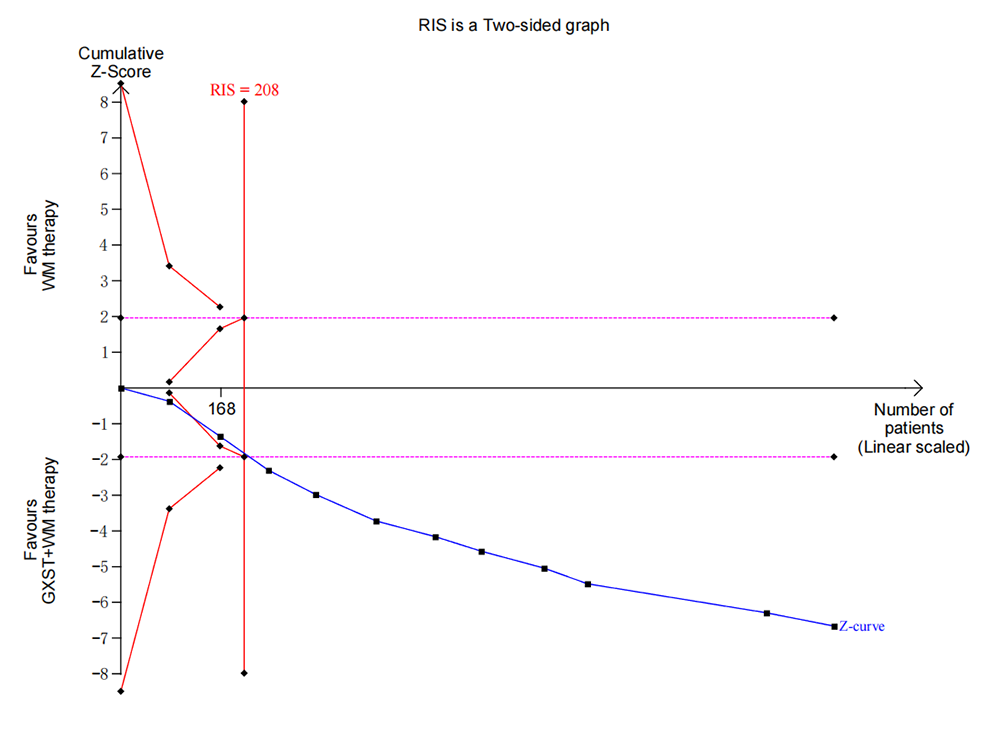
**

**The cumulative Z-curve of TSA**

**
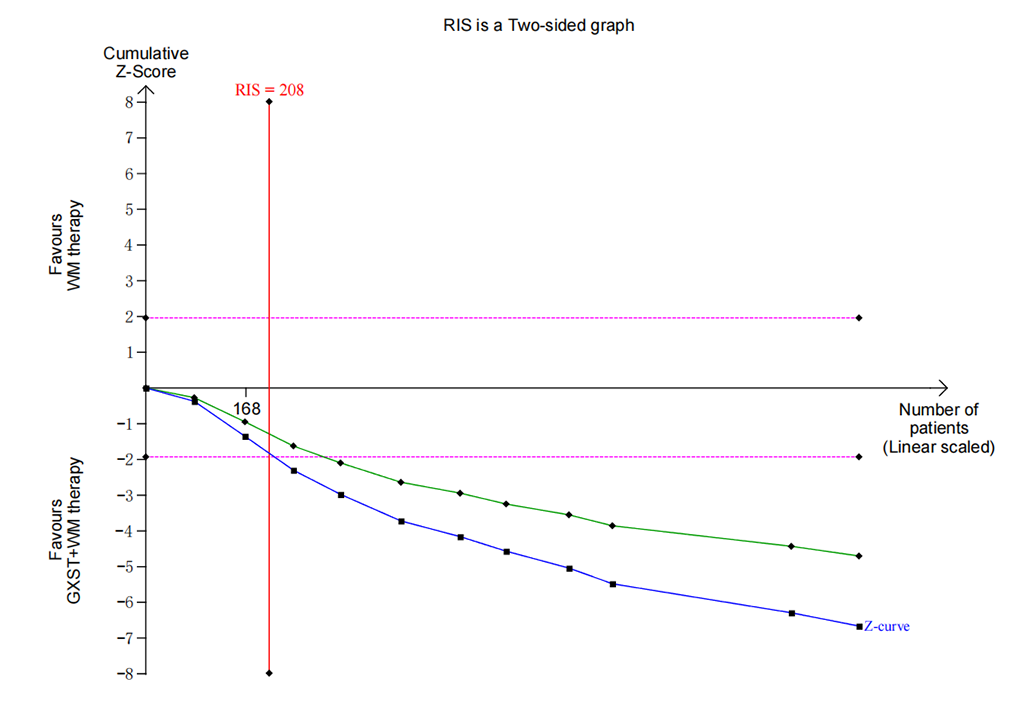
**

**The Z-curve of the penalty statistic of λ = 2**

**Figure S11.2 Trial Sequential Analysis of the effective rate in ECG**

**
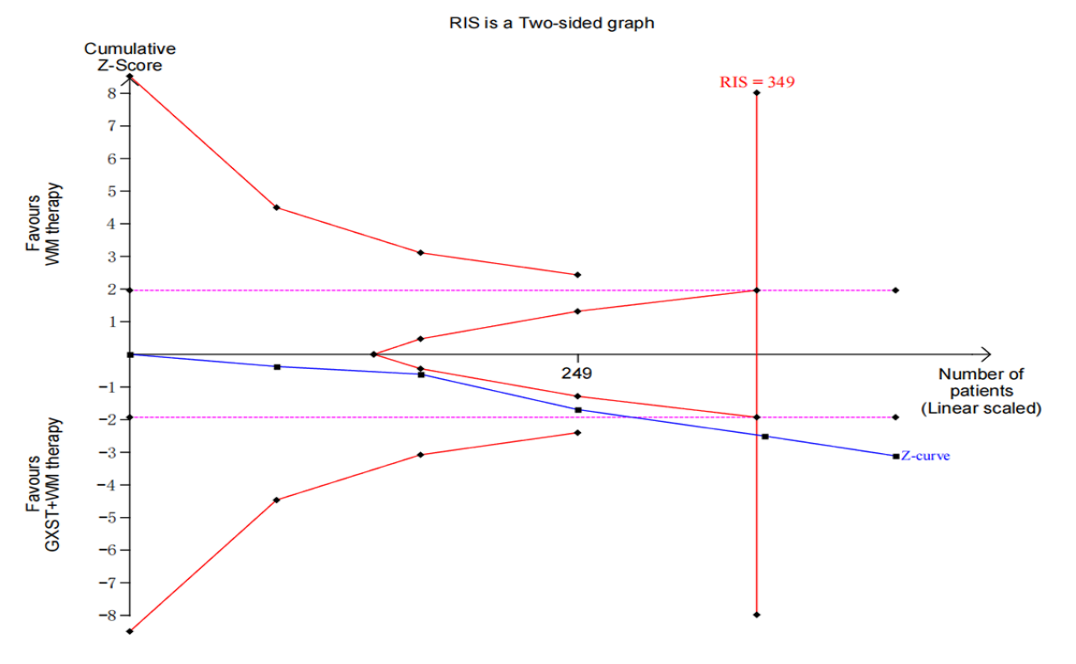
**

**The cumulative Z-curve of TSA**

**
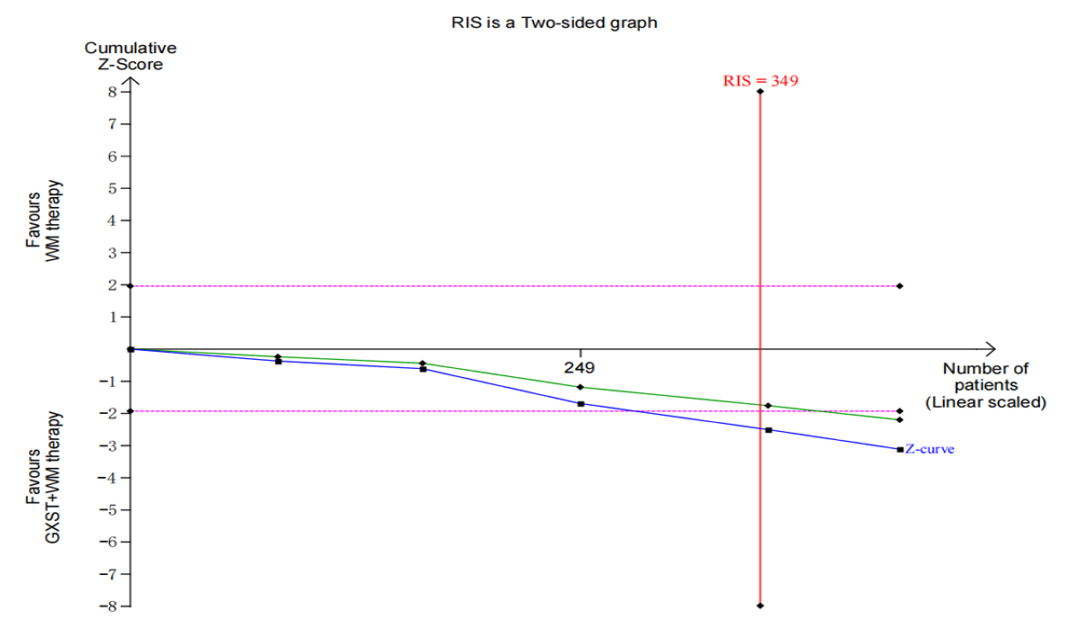
**

**The Z-curve of the penalty statistic of λ = 2**

**Figure S11.3 Trial Sequential Analysis of the frequency of angina pectoris**

**
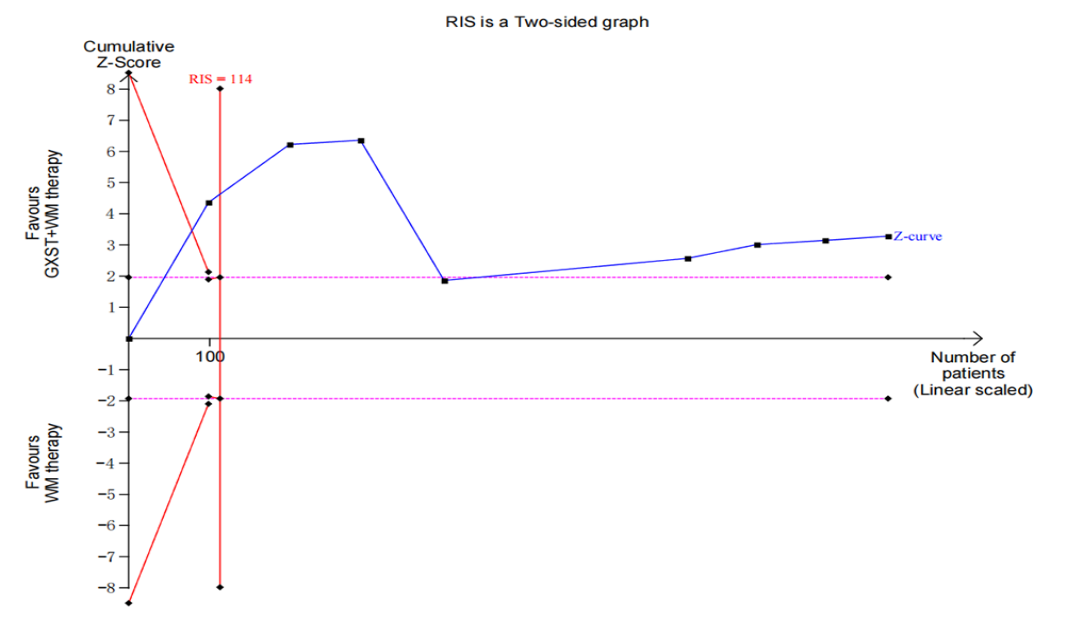
**

**The cumulative Z-curve of TSA**

**
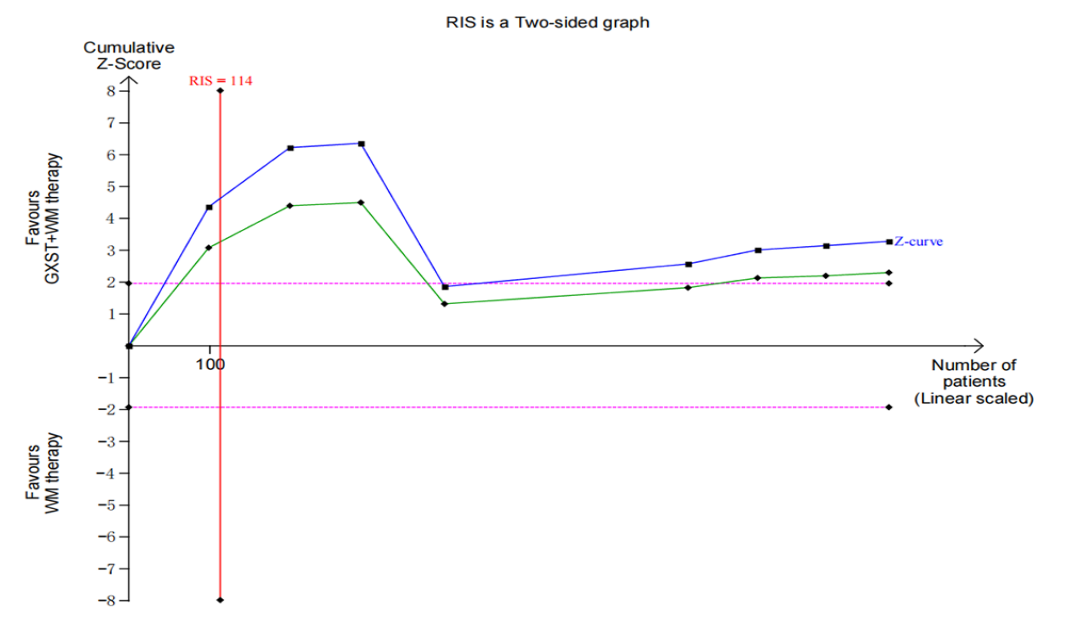
**

**The Z-curve of the penalty statistic of λ = 2**

**Figure S11.4 Trial Sequential Analysis of the duration of angina pectoris**

**
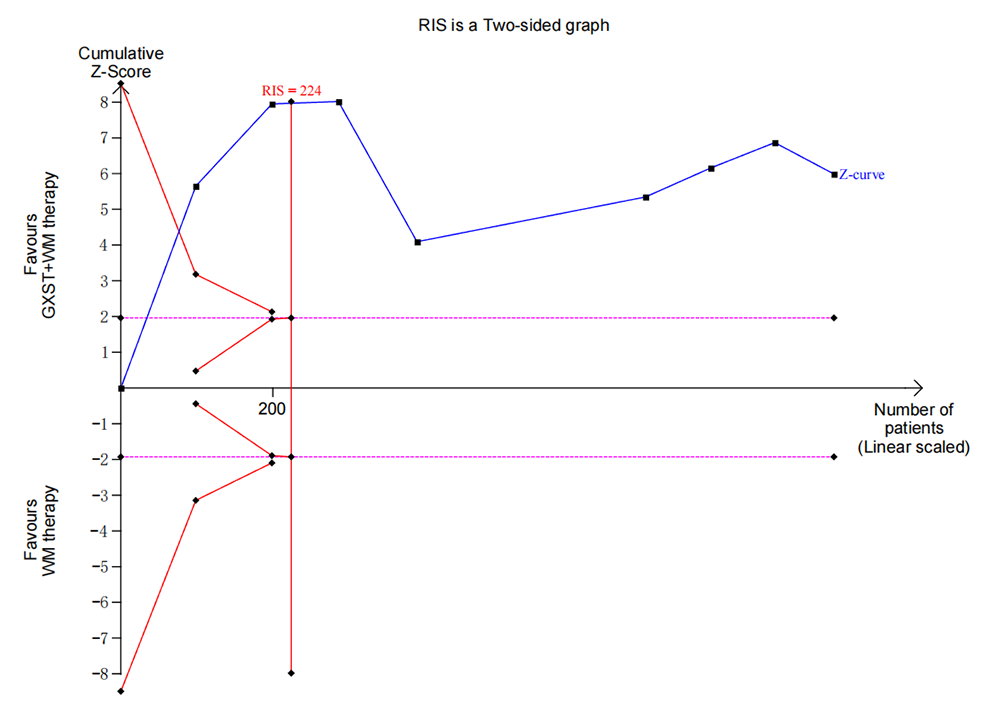
**

**The cumulative Z-curve of TSA**

**
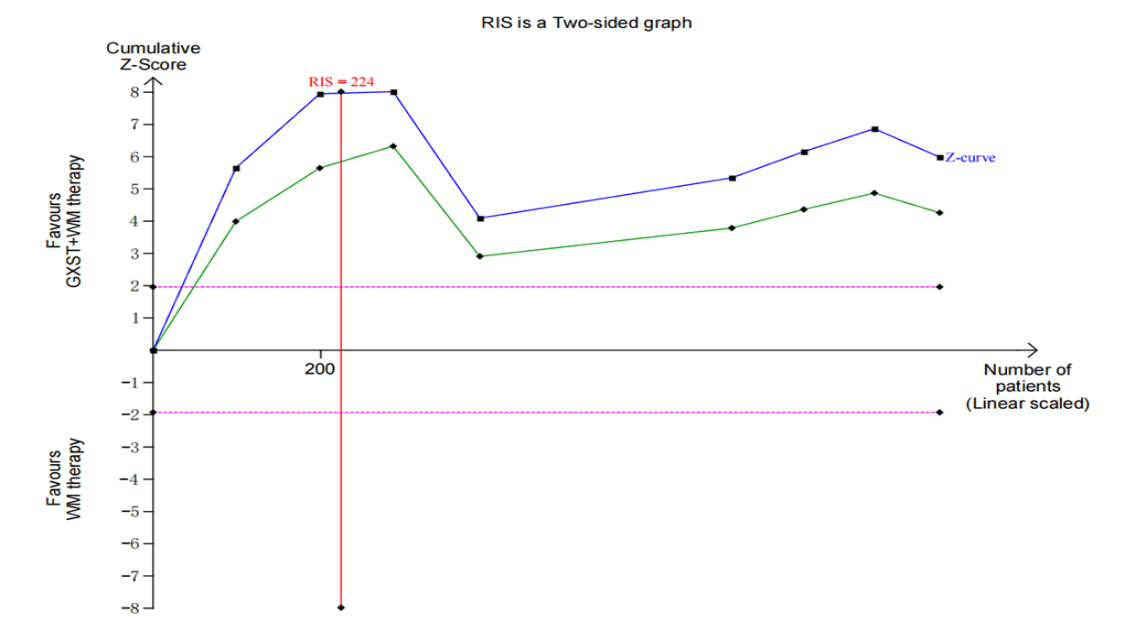
**

**The Z-curve of the penalty statistic of λ = 2**

**Figure S11.5 Trial Sequential Analysis of the adverse drug reactions incidence**

**
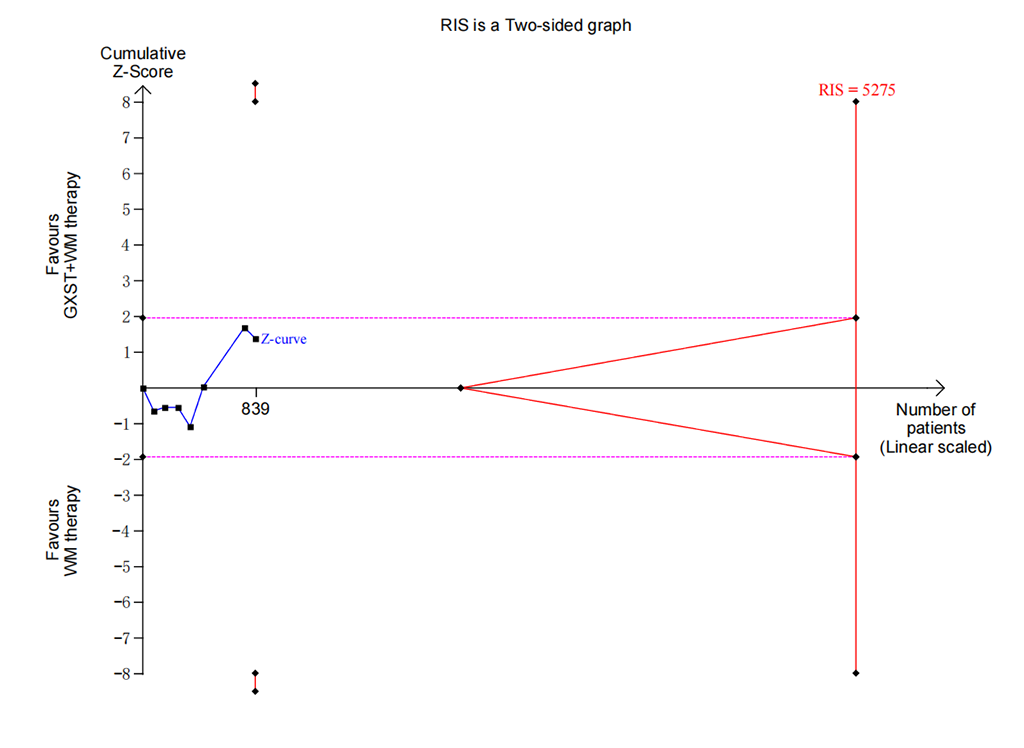
**

**The cumulative Z-curve of TSA**

**
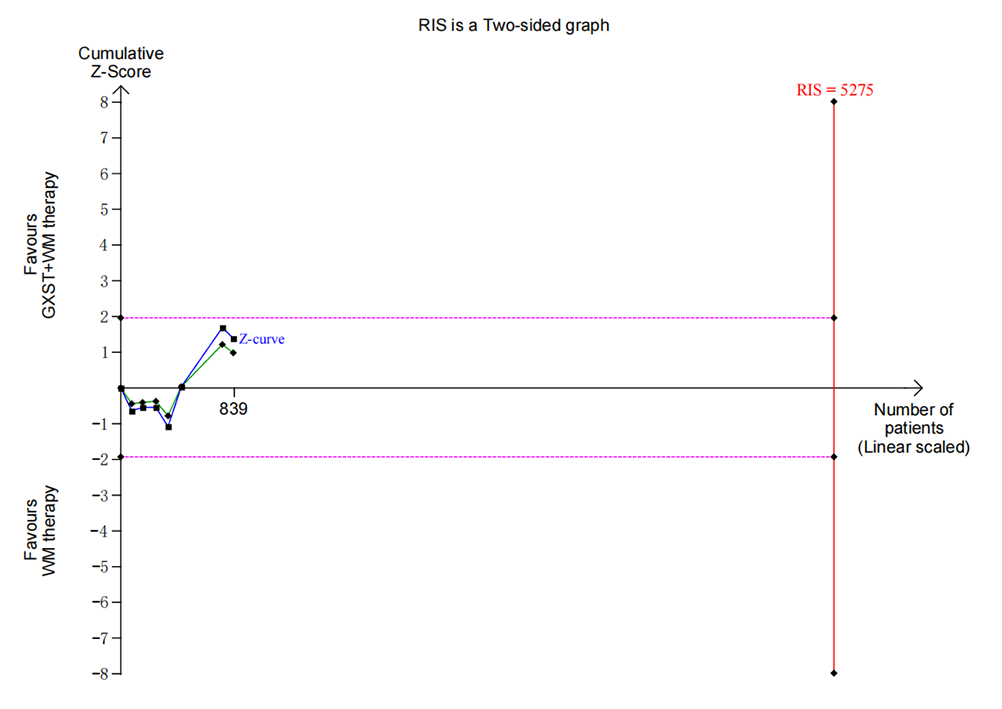
**

**The Z-curve of the penalty statistic of λ = 2**

**Appendix 12: GO enrichment analyses of drug-disease targets.**

**
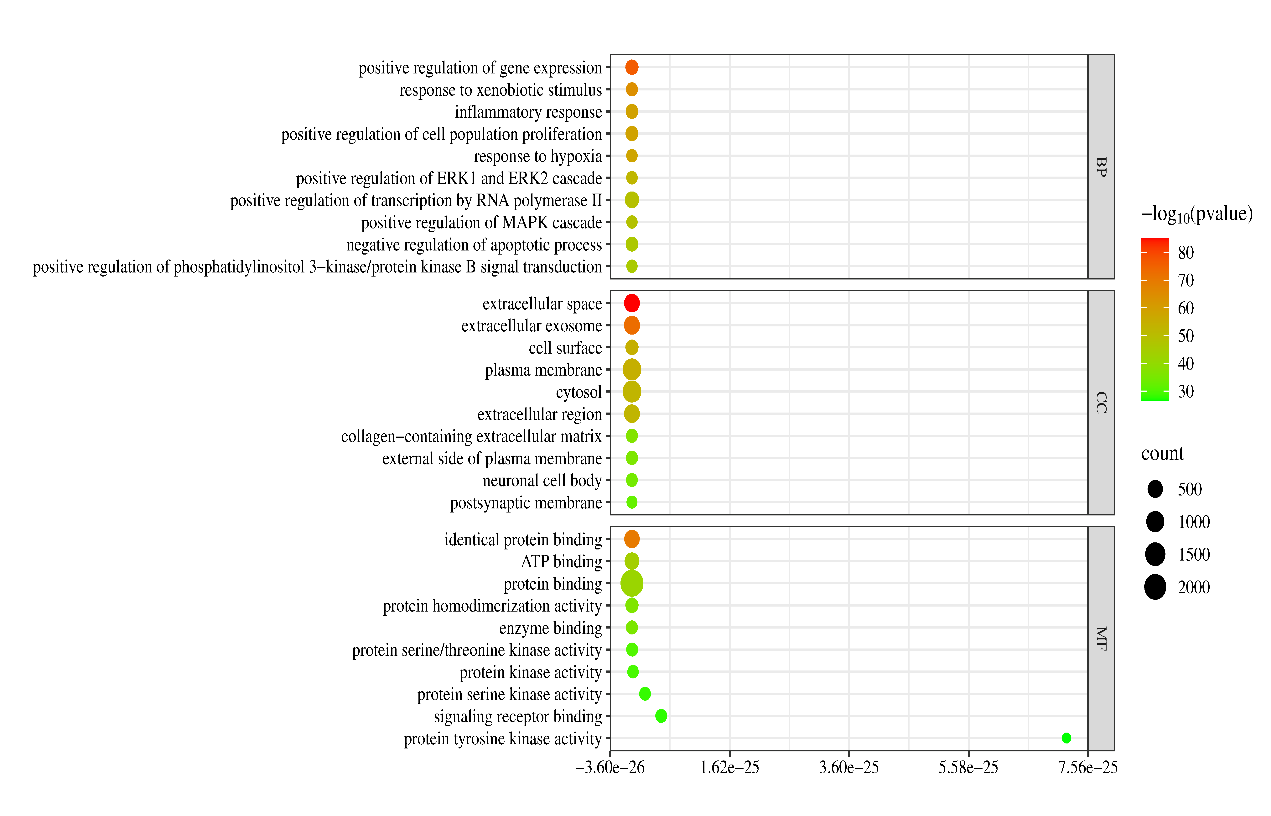
**

**Appendix 13: Top 10 hub genes in six algorithms.**

| Degree | Closeness | MCC | MNC | EPC | Radiality |
| --- | --- | --- | --- | --- | --- |
| HRAS | HRAS | HRAS | HRAS | HRAS | EGFR |
| PIK3CD | MAPK3 | PDGFRB | MAPK3 | MAPK3 | MAPK3 |
| TP53 | TP53 | JAK2 | PIK3CD | PDGFRB | SRC |
| EP300 | TNF | PIK3CD | TP53 | PIK3CD | TP53 |
| AKT1 | EP300 | TP53 | EP300 | TP53 | STAT3 |
| MAPK1 | AKT1 | EP300 | AKT1 | SMAD3 | EP300 |
| PIK3CA | MAPK1 | PTPN11 | MAPK1 | EP300 | AKT1 |
| KDR | PIK3CA | JAK1 | PIK3CA | AKT1 | MAPK1 |
| HSP90AA1 | ERBB2 | PIK3CA | HSP90AA1 | PTPN11 | JUN |
| PIK3CB | HSP90AA1 | IGF1R | PIK3CB | HSP90AA1 | INS |

**Abbreviations: MCC:** maximal clique centrality, **MNC:** maximum neighborhood component, **EPC:** edge percolated component
